# The Individual and Combined Effects of Warming and Atrazine on *Lithobates pipiens* Phenotypes: Implications for Frog Declines

**DOI:** 10.1002/jez.70054

**Published:** 2025-12-08

**Authors:** Melody J. Gavel, Mark R. Forbes, Derek D. N. Smith, Julia Darabaner, Yol Monica Reyes, Zintis Stasko, David J. Carpenter, Stacey A. Robinson

**Affiliations:** ^1^ Department of Biology Carleton University Ottawa Ontario Canada; ^2^ Ecotoxicology and Wildlife Health Division Wildlife and Landscape Science Directorate, Science and Technology Branch, Environment and Climate Change Canada Ottawa Ontario Canada

**Keywords:** amphibians, development, herbicides, immunity, pesticide, stress, temperature

## Abstract

Amphibians are the most threatened vertebrate class globally. Climate change, agrochemicals, and/or pathogens and parasites are implicated in contributing to amphibian declines, either singly or in combination. We investigated individual and combined effects of elevated temperatures and atrazine (2.0 μg/L) on *Lithobates* [formerly *Rana*] *pipiens* phenotypes, in a mesocosm experiment. We sampled tadpoles after 2 weeks, and other individuals at the completion of metamorphosis for endpoints relative to development, locomotor performance, immunity, and stress. Temperatures ranged from 7.18°C to 31.27°C over the experimental period, with a significant ~2°C difference between temperature treatments: warming and ambient. Whereas we found no effect of atrazine alone, we found strong effects of temperature, and some evidence of an interaction between atrazine and temperature on various phenotypic attributes. In tadpoles, elevated temperatures were associated with increased growth, accelerated development, and may have reduced stress, but decreased locomotor performance. Elevated temperatures also interacted with atrazine, offsetting an atrazine‐mediated delay in tadpole development. In metamorphs, elevated temperatures accelerated development at the cost of reduced size, but did not influence locomotor performance. However, warming was associated with lowered immunity, reflecting a trade‐off between growth and immune function. Elevated temperatures and atrazine also combined to affect metamorph neutrophil to lymphocyte ratios, reducing immunocompetency. Our results highlight the importance of incorporating multiple environmentally relevant stressors, thought important to amphibian declines in ecotoxicological studies, and of assessing multiple developmental stages.

## Summary

- We investigated the individual and combined effects of atrazine and warming on amphibians.
- Warming accelerated development, but reduced immunity.
- Atrazine alone had no effect, but it combined with warming to reduce immunity.

## 1 Introduction

Amphibians are declining globally (IUCN 2022). Population and species losses have been attributed to multiple causative agents, including climate change, and exposure to contaminants and/or to emerging infectious pathogens and parasites (Luedtke et al. 2023). Importantly, these factors may work independently, or in concert with each other, exacerbating amphibian population losses (Hof et al. 2011; Luedtke et al. 2023). In particular, exposure to contaminants such as agrochemicals can negatively affect amphibian immune systems, thus increasing their susceptibility to parasites (Kiesecker 2002; Rohr et al. 2008; Pochini and Hoverman 2017). Amphibians such as frogs are susceptible to potential toxic effects of agrochemicals because these chemicals find their way into surface water bodies that amphibians use for reproduction and development (Hamer and McDonnell 2008). Immunotoxicity can be a sub‐lethal effect in the absence of disease‐causing organisms (Brodkin et al. 2007). It is important to track such sub‐lethal effects to ascertain when and where amphibian losses are expected.

Atrazine is an agrochemical of relevant concern, as it is the second most common herbicide used in the United States (USEPA 2001). Atrazine is persistent and mobile in the environment and can enter surface waters through run‐off, spray‐drift, and by other means (Lin et al. 1999; Solomon et al. 1996). In 2007–2010, the atrazine concentrations in surface waters of Ontario, Canada ranged from 0.06 to 3.91 µg/L (Byer et al. 2011; Dalton et al. 2014); however, higher concentrations (> 20 μg/L), particularly during pulse inputs, have been historically reported in North America (reviewed in Solomon et al. 1996). As such, amphibians are at risk of exposure to atrazine, including during their sensitive developmental stages (Knutson et al. 2004).

Previous research has found that atrazine can act as an endocrine disruptor, feminizing frogs (Hayes et al. 2002). Further, atrazine can negatively affect amphibian survival, growth and development (Diana et al. 2000; Langlois et al. 2010). Atrazine also can have a negative effect on amphibian immunometrics (Brodkin et al. 2007; Hayes et al. 2006; Kiesecker 2002; Paetow et al. 2012) and can induce hyperactivity (Ehrsam et al. 2016; Rohr and Palmer 2005; Zhao et al. 2021). Notably, behavioral alterations are thought to be an early warning sign of toxicity in acute exposures, as behavior is often more sensitive to contaminants than other responses such as mortality (Hellou 2011). Amphibians must be able to respond to stimuli to avoid predation and parasitism (Eidietis 2006; Koprivnikar et al. 2006), and locomotor performance is important for basic survival in different ways such as foraging for food, dispersal/migration and predator evasion (Austin and Shaffer 1992). Whereas there is much literature regarding the concentrations at which atrazine exerts its effects on amphibians (reviewed by Hanson et al. 2019; Luedtke et al. 2023; Rohr and McCoy 2010), there is a dearth of information on if these, or lower, concentrations of atrazine interact with other factors such as temperature to affect phenotypes of amphibians.

In addition to exposure to agrochemicals, climate change‐driven shifts in surface water temperatures are expected to affect amphibians. Climate change is predicted to increase surface water temperatures (Magnuson et al. 1997; Smith et al. 2021). Globally, surface temperatures are predicted to increase by 1.0°C–5.7°C by the year 2100 (IPCC 2023). In Canada, within the Great Lakes region (including nearby streams and wetlands), temperature simulations predict an increase of between 1°C and 7°C in surface waters (Magnuson et al. 1997). More broadly, mean surface water temperatures in Ontario lakes are predicted to increase by 3.8°C–6.7°C by 2080 (Smith et al. 2021). As amphibians are ectotherms, these changes in water temperature are likely to influence their physiology, including their immune systems (Blaustein et al. 2010; Maniero and Carey 1997). Specifically, compared to cool hibernating temperatures, warmer, seasonally relevant temperatures, can upregulate amphibian immunity (Cooper et al. 1992; Maniero and Carey 1997; Matutte et al. 2000); however, chronic exposure to unseasonally warm temperatures can also act as a stressor to amphibians, resulting in immunosuppression (Lima et al. 2020; Weerathunga and Rajapaksa 2020), and increased susceptibility to parasites and pathogens (Lima et al. 2020). For example, Weerathunga and Rajapaksa (2020) found that warmer temperatures reduced *Polypedates cruciger* immunity, locomotor performance, and development. Few studies have been conducted to look at the effects of chronically elevated temperatures on amphibian immunity, both independently, or in concert with exposure to agrochemicals (Blaustein et al. 2010; Rollins‐Smith and Le Sage 2023). As temperatures in North America are predicted to rise given climate change, this marks an important avenue for continued research (Noyes et al. 2009).

The interactive effects of pesticides and elevated temperatures on amphibians can be contrasting. For example, elevated temperatures can increase the amphibian's rate of development, thus reducing their exposure duration to water‐borne pesticides (Rohr et al. 2011). However, elevated temperatures also can increase metabolic rate, thereby increasing the uptake of pesticides and increasing their toxicity (Hooper et al. 2013). There is a significant knowledge gap regarding the combined effects of atrazine and elevated temperatures on amphibian immunometrics. Pathogens and parasites play a role in the global decline of amphibians and may be exacerbated by interactions between other sub‐lethal stressors such as contaminants and temperature (Blaustein et al. 2018). As such, an investigation into the combined effects of a common herbicide and elevated temperatures on amphibian immunity is warranted. Furthermore, the combined effects of atrazine and warmer temperatures on the life‐history characteristics of different species should be investigated because different amphibian species can have differing responses to environmental conditions (e.g., pesticides and temperature; Lau et al. 2015; Ujszegi et al. 2022).

The objectives of our study were to assess the effects of experimentally elevated temperatures and contaminant exposure, both individually and in combination, on the development, locomotor performance, immune systems, and stress of northern leopard frogs (*Lithobates pipiens;* formerly *Rana pipiens*) at two stages of development (see Methods and Supporting Information S1: Table 1 for full breakdown of metrics measured by life history stage).

## 2 Methods

### 2.1 Experimental Design

**Figure 1:**
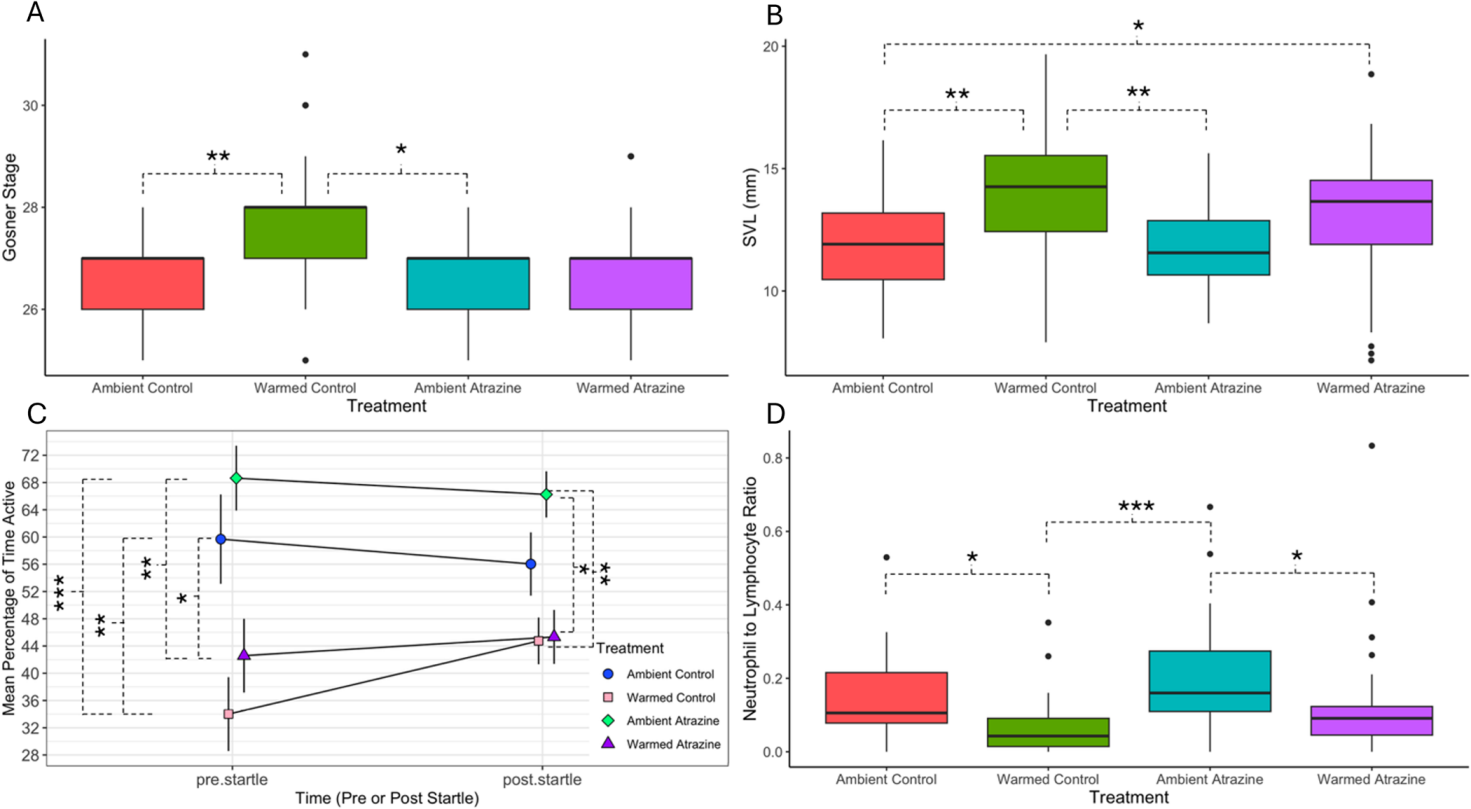
Plots for *Lithobates pipiens* tadpole endpoints assessed after 2 weeks of exposure to warmed/ambient temperatures and atrazine/control water. (A) Gosner stage of development among treatments. (B) Snout to vent length (SVL; mm) among treatments. (C) Mean percentage of time active among treatments, before and after a startle. (D) Neutrophil to lymphocyte ratios among treatments. Significant differences (*p* < 0.05) are denoted by dashed braces, with asterisks denoting significance level (*p < 0.05, **p < 0.01, ***p < 0.001). For boxplots A, B and D, the dark horizontal lines show the median, while the large rectangles above/below these bars represent the 25% and 75% interquartile ranges. The vertical lines above these rectangles show 1.5 times the difference between the interquartile ranges. Outliers are shown by black dots. For plot C, dots represent the mean, and the lines above and below the dot represent the standard deviation. For the locomotor performance plot (C), only within interval significance is shown. For full significance breakdown, see Supporting Information S1: Tables 6 and 8.

We conducted a 2 × 2 factorial experiment using an ecologically relevant concentration of atrazine (2 μg/L) and experimental warming of water versus no warming in mesocosms. There were four, fully factorial treatments: an ambient water control, warmed water, and ambient and warmed water with nominal concentrations of 2 µg/L of atrazine. We included five replicates of each treatment, spread across five blocks in randomized order (Supporting Information S1: Figure 1). This design aimed to simulate current versus future thermal regimes. The ambient control treatment served as a baseline, while the ambient atrazine treatment reflected current exposure and temperature scenarios. The warmed control treatment simulated a future scenario wherein atrazine is phased out of use, whereas the warmed atrazine treatment reflected a scenario with continued atrazine use and elevated temperatures.

The atrazine concentration (2 μg/L) was selected to represent concentrations measured in the environment (e.g., 0.06–3.91 µg/L; Struger et al. 2004; Byer et al. 2011), and concentrations that have been shown to have immunotoxic effects on amphibians (e.g., 0.001 μg/L and 160 μg/L; Brodkin et al. 2007; Forson and Storfer 2006). Furthermore, this concentration is close to the Canadian Council of Ministers of the Environment (CCME) guidelines for the protection of freshwater life (1.8 µg/L; Canadian Council of Ministers of the Environment CCME 1999), while still being relevant to concentrations of atrazine found in the environment. Finally, we ensured the concentration was below a threshold concentration, which was previously found to increase mortality in our study species (Langlois et al. 2010).

*Lithobates pipiens* is a common native species found throughout the Eastern Ontario region (and across Canada) and is an early spring breeder (Green and Taylor 2009). In the wild, these frogs can be exposed to contaminants in surface waters that are warming due to climate change. We aimed to elevate water temperatures by 3°C–4°C, which is within the range of predicted temperature increases where *L. pipiens* are found, to ensure our study remained relevant to current climate projections.

We assessed fitness relevant measures at two stages of development, with *L. pipiens* tadpoles sampled after 2 weeks of exposure to atrazine and/or warm temperatures and individuals sampled once they completed metamorphosis (stage 46; Gosner 1960). It is important to assess the effects of contaminants at multiple stages of amphibian development, given that some life stages may be more/less susceptible to the effects of contaminants (Mitchkash et al. 2013). We selected two sampling periods as they represent vulnerable windows in the frog's development. Specifically, tadpoles in the early stages of development have immature immune systems and may be more susceptible to pathogens (Rollins‐Smith and Woodhams 2012). In comparison, metamorphosis involves the complete reorganization of the immune system and many biochemical changes, which can be affected by exposure to pesticides (Rollins‐Smith and Woodhams 2012; Boone et al. 2013).

Endpoints were selected to encompass three broad categories: development, locomotor performance, and stress/immunity, to capture a snapshot of overall ‘health’ of tadpoles or metamorphs. Beyond mortality, we assessed both life history stages for sub‐lethal developmental endpoints including size and development rate. These sub‐lethal fitness measures are important to quantify, as they can have downstream fitness‐related consequences for amphibians (Székely et al. 2020; Ward‐Fear et al. 2010). It is important to consider that atrazine and warming temperatures may each have opposing effects on these metrics (Brodeur et al. 2013; Freitas et al. 2017; Weerathunga and Rajapaksa 2020), and when combined, can interact negatively (Rohr et al. 2011).

We further assessed both tadpoles and metamorphs for their locomotor performance and startle response. Startle response was measured as time to movement following a standardized stimulus, and locomotor performance was measured by swim speed in tadpoles and hop distance in metamorphs. We also assessed both life history stages for endpoints relating to immunity and stress. Specifically, both tadpoles and metamorphs were assessed for their leukocyte profiles. Metamorphs were further assessed for their blood glucose concentrations and the bacteria‐killing ability (BKA) assays of their blood plasma. Leukocyte profiles are a commonly used metric for assessing stress in vertebrates (Davis et al. 2008). Namely, increases in the ratio of neutrophils, relative to lymphocytes (NL ratio), can be indicative of a higher stress level (Davis et al. 2008). Unlike other measures of stress such as corticosterone, NL ratios do not attenuate over time (Davis and Maney 2018). As chronic stress can increase susceptibility to parasites (Belden and Kiesecker 2005; LaFonte and Johnson 2013), NL ratios can provide insight as to the susceptibility of the organism to disease. In addition to NL ratios, blood glucose can also be used as a secondary indicator of stress (Broughton and deRoos 1984). Specifically, blood glucose levels rise with stress in amphibians (Dias dos Santos et al. 2021; Harri 1981). Finally, plasma BKA incorporates complement, antibodies, and lysozymes (Rodriguez and Voyles 2020) and this provides an integrated measure of immunocompetence. Environmental factors often have variable effects on different components of the stress response and immunity (Brannelly et al. 2019; Weerathunga and Rajapaksa 2020). As such, we include multiple measures of stress/immunity, to provide a more comprehensive assessment.

### 2.2 Experimental Set‐Up

The experiment was conducted in a designated research area at Carleton University, Ontario, Canada. We used 20 Rubbermaid cattle tanks (132 × 78 × 63 cm; henceforth “mesocosms”), and buried the bottom 23 cm of each mesocosm to help reduce variation in water temperature, which also can affect amphibian immunity (Raffel et al. 2006). All experimental materials, including mesocosms were first washed with Liquinox soap, triple rinsed with tap water, washed with 10% bleach, and triple rinsed again, and then allowed to air dry. Mesocosms were lined with TotalPond PVC liners (safe for aquatic life). Mesocosms were aerated using bubblers set to a gentle air stream with an air stone. HOBO data loggers and digital aquarium thermometers (AxGear) were secured four inches from the bottom of the mesocosm using four‐inch PVC tubing. Aquatic cages (58 L, described in Harris et al. 2001) were affixed inside of each mesocosm using plastic fencing.

**Figure 2:**
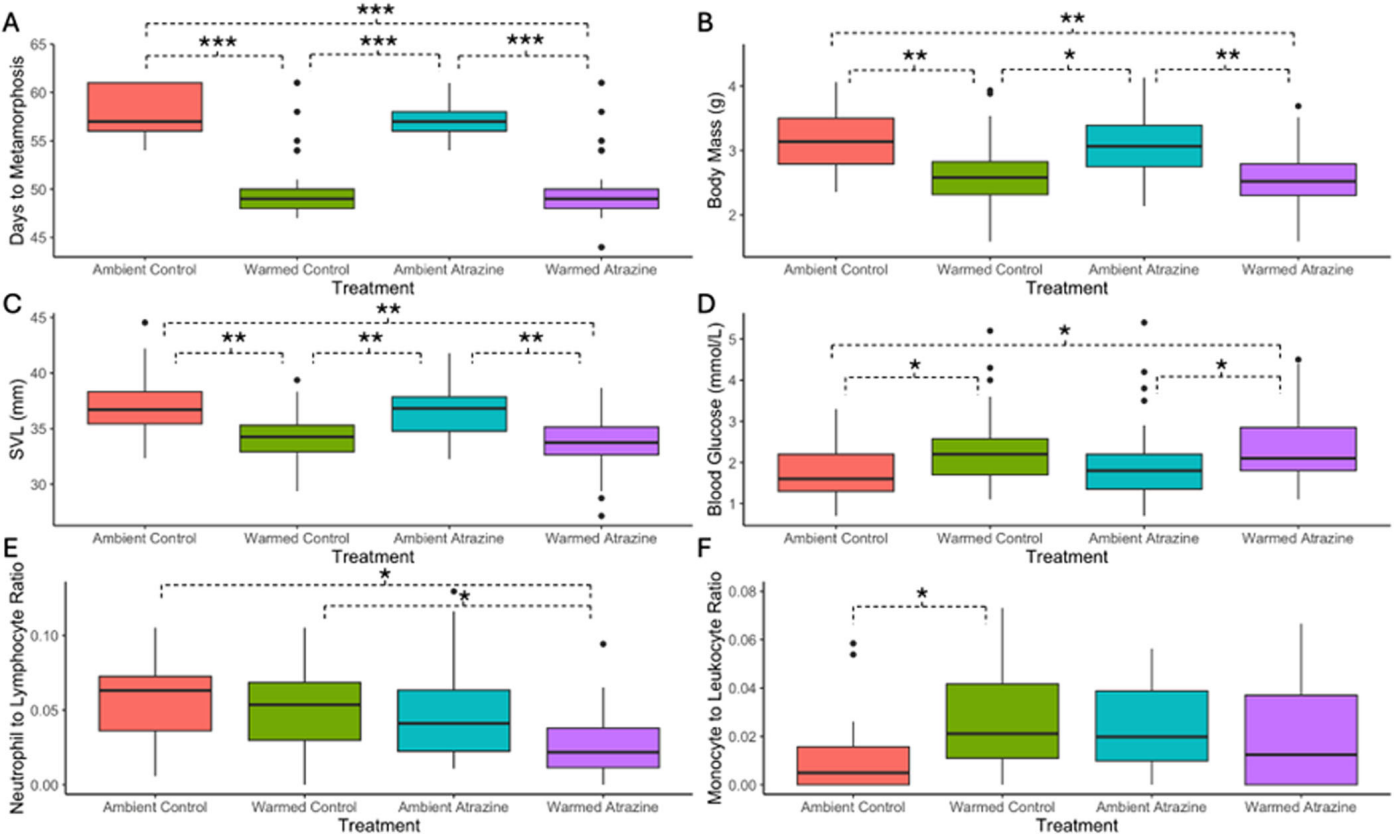
Plots for *Lithobates pipiens* metamorph (stage 46) endpoints assessed after exposure to warmed/ambient temperatures and atrazine/control water. (A) Days to metamorphosis among treatments. (B) Metamorph body mass (g) among treatments. (C) Metamorph snout to vent length (SVL; mm) among treatments. (D) Blood glucose (mmol/L) among treatments. (E) Neutrophil to lymphocyte ratios among treatments. (F) Monocyte to leukocyte ratios among treatments. Significant differences (*p* < 0.05) are denoted by dashed braces, with asterisks denoting significance level (*p < 0.05, **p < 0.01, ***p < 0.001). The dark horizontal lines show the median, while the large rectangles above/below these bars represent the 25% and 75% interquartile ranges. The vertical lines above these rectangles show 1.5 times the difference between the interquartile ranges, and black dots indicate values outside this range.

Mesocosms were filled with 200 L of City of Ottawa tap water treated with Seachem Prime® to neutralize both chlorine and chloramine. Greenhouses (162.56 × 109.22 × 160.03 cm; Supporting Information S1: Figure 2) were placed atop of each mesocosm. Ambient treatment greenhouses only had plastic roofing, whereas warmed treatment greenhouses were fully covered in plastic. We included the roof‐only greenhouses over ambient treatments to prevent any differences due to rainfall, and rainwater chemical composition. All mesocosms were covered with 40% shade cloth to prevent entry of any unwanted organisms. When tadpoles began to develop rear limbs, we added floating rafts (20 × 20 cm high‐density polyethylene; Dalton et al. 2014) to each mesocosm to provide resting areas.

We used lab‐cultured microbiota to limit possible growth of pathogens into the mesocosms (according to Hamilton et al. 2012). We added 500 mL of *Chlorella vulgaris* culture to each mesocosm 2 weeks before the start of the experiment, and 500 mL of *Daphnia magna* culture to each mesocosm 1 week before the start of the experiment to support the development of the aquatic environment. We also inoculated each mesocosm with 33 g of Organic Bounty organic rabbit pellets (according to Hamilton et al. 2012).

### 2.3 Northern Leopard Frog Husbandry

All animal handling and care was done in accordance with the Canadian Council for Animal Care guidelines and was approved by the governing animal care committees: Environment and Climate Change Canada's Wildlife East Animal Care Committee (23SR01) and the Carleton University Animal Care Committee (119458).

Three freshly spawned *L. pipiens* egg masses were collected from a non‐agriculture site in the National Capital Region, Ontario, Canada on May 2, 2023. The eggs were brought to the University of Ottawa for overnight storage, before being transported to Carleton University on May 3rd. The egg masses were kept in a Conviron environmental chamber set to 21°C and 70% humidity, on a 12‐h light/dark cycle. The egg masses were separated into three separate 45 L tubs, each containing 23 L of aged, dechloraminated water where eggs hatched 7 days later (May 9th). Once tadpoles were free‐swimming, we commenced 50% water changes three times weekly, and fed the tadpoles Ward's Xenopus tadpole food and President's Choice organic frozen kale after each water change, ad libitum. On Fridays, the tadpoles were also given algae wafers (Specialist's Fish Food Hikari Tropical).

On May 16th, we randomly selected 20, 20, and 21 tadpoles from each of the three tadpole stock tanks, such that the combined total per mesocosm was 61 tadpoles. The stock tank providing 21 tadpoles was alternated for each selection. One additional tadpole was added to each mesocosm for use in a separate study. We used tadpoles from the three separate egg masses to introduce genetic diversity, meeting the minimum recommended number for representing genetic variation, consistent with established practices in amphibian ecotoxicology research (Boone and James 2003). The tadpoles were visually estimated to be stage 25, characterized by the absence of external gills and limb buds (according to Gosner 1960). We added the tadpoles to mesh cages in a randomized block order. Mesh cages were used to facilitate subsampling of tadpoles after 2 weeks of exposure. Tadpoles were released from their mesh cages into the mesocosms on June 2nd (experimental day 16). We quantified survival of all tadpoles (i.e., the 21 sampled and the remaining 40 per mesocosm) during the release of the tadpoles on June 2nd, and then removed the mesh cages from the mesocosms. Through non‐invasive observation, we visually monitored tadpole development, mesocosm water levels and tadpole welfare (according to Canadian Council on Animal Care CCAC 2021a) three times weekly during feeding. However, as some of the tadpoles in the mesocosms approached stage 46 in development (June 28th), we monitored tadpole development more frequently to ensure endpoints were collected from recently metamorphosed frogs. To maintain water quality during this period of staggered development, feces were removed from tanks on June 21st and 28th. To maintain water levels, we also added 10 L of temperature‐specific (ambient or warmed) water that had been aged and treated with Seachem Prime® to remove chlorine and chloramine to each mesocosm on June 28th.

### 2.4 Atrazine Herbicide

We combined 18.75 µL of the commercial formulation of atrazine, Aatrex Liquid 480 (480 g/L, Syngenta Canada Inc. Registration number: 18450) and 0.9 L of deionized water to prepare a concentrated stock solution (nominal concentration 10,000 µg/L). We used the commercial formulation to reflect environmentally relevant exposure scenarios (i.e., to reflect what is applied in agricultural settings). The stock solution was stored in an amber glass bottle and kept at 4°C in the dark until use. On May 17th, the atrazine concentrated stock solution was added to the herbicide‐treatment mesocosms, for a nominal concentration of 2 µg/L (experimental day 0). Non‐herbicide treatment mesocosms received the same volume of deionized water. We collected water samples from each mesocosm 1 h after dosing to confirm nominal concentrations and subsequently collected from all block three mesocosms weekly to monitor atrazine concentrations throughout the experimental period. We also collected water samples from every mesocosm on May 30th (experimental day 13), and on the final day of the experiment (July 17th, experimental day 61). For full detailed methodology surrounding atrazine sample collection and analysis, refer to Supporting Information.

### 2.5 Physicochemical Water Quality

We monitored dissolved oxygen, pH, temperature, and conductivity using a YSI (YSI Professional Plus) on a weekly basis from block three of the experimental mesocosms. The YSI temperature readings were used in conjunction with pH readings (collected at the same time) to calculate unionized ammonia levels. To determine if aquatic conditions remained within guidelines (Canadian Council on Animal Care CCAC 2021b; OECD 2009, 2015) we monitored ammonia, nitrate, and nitrite using an API freshwater Master Test Kit (Mars Fishcare North America Inc). However, as our test kit measured only total ammonia, unionized ammonia was calculated according to Francis‐Floyd et al. (1990). We also monitored water hardness using an API GH and KH Test Kit (Mars Fishcare North America Inc). For daily temperature monitoring, we used axGear electronic digital water thermometers, outfitted in each mesocosm. We recorded water temperature from each mesocosm twice daily on weekdays, and once daily on weekends. This monitoring was done to determine if mean temperature differentials between warmed and ambient mesocosms remained around 3°C, while not disturbing the tadpoles. When the temperature differential surpassed 3°C or water temperatures approached 28°C, we vented warmed greenhouses by removing insulating plastic strips from greenhouse lids and cut a hole on the vertical sides of the warmed greenhouses to allow excess heat to escape to deal with rising seasonal temperatures. For in‐depth temperature measurements (used in statistical analyses), we used HOBO (HOBO – Onset – UA‐002‐64 pendant) data loggers in each mesocosm to monitor temperatures on an hourly basis.

### 2.6 Tadpole Endpoints

On experimental days 13–15 (May 30th to June 1st), we subsampled a total of 21 tadpoles from each mesocosm, in randomized block order, such that one or two blocks were processed each day. Of those 21 tadpoles per mesocosm, eight were assessed for their locomotor performance and startle response using a standardized startle assay (Supporting Information S1: Figure 3), 12 were assessed for leukocyte differentials, and one was euthanized and stored for future transcriptomics research (not included in this study). We did not include the tadpoles assessed for locomotor performance in the leukocyte endpoints since the startle assay had the potential to influence these metrics. Finally, all tadpoles were assessed for apical endpoints (stage of development/size).

Tadpoles in the locomotor performance group completed assays prior to apical measurements, to minimize any influence of handling stress. We developed the standardized startle assay as a modification of do Amaral et al. (2018) and Krishna and Anirudha (2020). Krishna and Anirudha (2020) used a tap on the glass of tadpole aquariums to determine whether a startle response was present and monitored movement before and after startle. As we were unable to monitor tadpole movement reliably in the mesocosms, we modified the startle technique.

To perform tadpole locomotor performance trials, we built a startle assay that dropped a metal bar from a height of 30 cm. We used black cardboard dividers to ensure tadpoles could not see each other and built a large black box around the startle assay to mimic mesocosm conditions and to minimize any influence of activity outside of the startle arena. We kept each tadpole in 200 mL of dechloraminated water, at the same temperature as their respective outdoor mesocosms, using water from additional mesocosms, outfitted with either a full or vented greenhouse. We controlled for the water temperature to avoid the effect of thermal stress. We used a GoPro Hero 8 to film the tadpoles for the entirety of the startle assay.

We assayed one tadpole from each treatment at the same time and allowed tadpoles to acclimate within the startle assay arena for 2 min before beginning filming. From there, we filmed the tadpoles for 5 min before activating the bar‐dropping mechanism, which produced auditory, vibrational and visual stimuli. We then filmed the tadpoles for an additional 5 min. The position of the tadpoles within the startle assay mechanism was randomly determined using Random.org. Our startle response assay was designed to deliver a controlled and consistent stimulus simultaneously to a representative tadpole from each treatment group, while minimizing handling stress and ensuring reproducible measurements, key factors in reducing variability in behavioral responses. We analyzed the tadpole videos completely blind, to ensure no bias. We recorded whether a tadpole was active at every 10 s interval, to then calculate the percentage of time the tadpole was active before and after the startle. We also assessed whether the tadpole moved in response to the startle.

We visually estimated the developmental stage of the 21 tadpoles per mesocosm. We photographed the tadpoles over graph paper using a Sony (Cybershot DSC‐RX100) camera for snout to vent length (SVL) measurements. We measured tadpole SVL using ImageJ software (version 1.54 d). We then calculated the precision of SVL measurements by calculating the mean coefficient of variation across three randomly selected tadpoles, by measuring these tadpoles on 20 separate occasions throughout the measurement timeframe without reference to previous measurements. The mean coefficient of variation was 0.01%, which is within an acceptable level of precision according to Hayek et al. (2001).

We collected blood smears by first anesthetizing the tadpoles in 0.02% buffered MS‐222 (tricaine methane sulfonate; Sigma‐Aldrich), and then euthanizing the tadpoles using 0.20% MS‐222. We then collected blood smears according to Gavel et al. (2019). Briefly, we dissected out the tadpole's heart and smeared it in a U‐shape along a glass slide. Once dry, we fixed the slide in 100% methanol and then stored it for future enumeration.

Tadpole blood smears were stained using modified (0.4% w/v methanol) Wright‐Giesma stain and enumerated according to Gavel et al. (2019). Briefly, we first scanned the blood slide at 10X objective using a VanGuard light microscope (model ISH1000) to assess the overall blood density—that is, identify areas of the blood smear where cells were not too sparse or overlapping. From there, we focused in on the area of the slide most representative of the blood smear cell density. This method assured we used fields of view that represented the smear as a whole, and not areas where blood may have pooled or been very sparse on outer edges of the smear. We then used the 40X objective and a TUCSEN camera (model ISH1000; additional magnification of 0.5X) to photograph 100 fields of view using TCapture software (version 5.1.1.0) from each blood smear, ensuring no overlapping fields of view were photographed. We then identified and counted blood cells in each field of view according to Gavel et al. (2019) using Hadji‐Azimi et al. (1987) as an identification guide. Evaluation of the blood smears were completed blind to ensure no accidental bias.

### 2.7 Metamorph Endpoints

When frogs reached stage 46 (completion of metamorphosis, experimental days 44‐61; henceforth called metamorphs), these metamorphs were removed from mesocosms for processing. The first 20 metamorphs to complete metamorphosis were divided into one of two endpoint processing groups. We allocated 10 metamorphs to be assessed for locomotor performance, and the other 10 metamorphs to be used for dissection, assigning odd numbered metamorphs to the locomotor assessment group, and even numbered metamorphs to the dissection group. This division ensured an even distribution of metamorphs across the processing days. When 20 metamorphs from each mesocosm completed metamorphosis (July 17, experimental day 61), the experiment was terminated. Every metamorph to reach stage 46 by experimental day 61 (including those not included in locomotor/dissection endpoints) had days to metamorphosis calculated, and any remaining tadpoles that did not reach stage 46 by experimental day 61 had their stage visually estimated and recorded.

Locomotor trials were conducted in a temperature‐controlled greenhouse room, with the ambient temperature set to the daily outdoor temperatures. This temperature control ensured that locomotor trials were conducted at similar temperatures to what the metamorphs were experiencing and reduced the potential effects of thermal shock. To assess locomotor performance, we modified methods from Zug (1975, 1985) and Thompson et al. (2022) (see Supporting Information for full breakdown of metamorph locomotor trials and photo of locomotor arena; Supporting Information S1: Figure 4). We allowed the metamorph to jump 11 times. If the metamorph did not jump after 5 s, it was gently prodded in the urostyle using a paintbrush. If the metamorph still did not jump after three prods, the assay was terminated (i.e. “Fail;” in accordance with Thompson et al. 2022). We used an ACEGMET laser measurer (accuracy +/− 2 mm) to measure the distance between each jump. We did not include the first jump in our analyses as the first jump can represent acclimation to the metamorphs’ new environment according to Zug (1972). We did not perform repeated trials on individual metamorphs, in order to minimize handling stress, which could affect locomotor performance (according to Zug 1972). The assays were conducted in a quiet, undisturbed room, and the technician completing the assay remained at the starting side of the arena. Locomotor trials were conducted in a randomized block order (i.e., for a given block, all individuals from one tank were assessed, followed by the next tank in the randomly determined order, until all individuals to have completed metamorphosis in that block were assessed—this process was repeated for each block).

Following locomotor performance trials, metamorphs were anesthetized in 0.02% MS‐222, euthanized in 0.2% MS‐222, and then photographed over graph paper for future measurement of SVL, femur length, and tibiofibular length (according to Moreno‐Rueda et al. 2020 and Petrović et al. 2017; Figure S5). The coefficient of variation, as described above for tadpoles, was calculated for each of these measures, and was 0.01%, which is within acceptable levels of precision (Hayek et al. 2001).

Metamorphs in the dissection group were first anesthetized in 0.02% MS‐222, patted dry using a kimwipe, and then weighed using a Mettler Toledo MS204TS scale. The metamorph was then euthanized in 0.2% MS‐222. Immediately following euthanasia, the metamorph had blood collected from the abdominal vein for future BKA assays according to Owen et al. (2018). We also measured blood glucose levels using a Contour Next One glucose meter (Lot DW01R308P). Blood samples collected for BKA assays were kept on ice for no more than 2 h before being centrifuged (Eppendorf Centrifuge Model 5430 R) at 3500 rpm for 10 min to isolate and collect plasma. The plasma was then flash frozen in liquid nitrogen and stored at −80°C. Following blood collection, the metamorph was dissected to collect a blood smear (as previously described in tadpole week 2 endpoints). We then dissected out and weighed the liver using a Mettler Toledo MS204TS scale. The hepatosomatic index was calculated using the following formula:

$$\left(\left(\frac{\mathrm{liver mass}}{\mathrm{body mass}-\mathrm{liver mass}}\right)\;*\;100\right)$$

Here, the hepatosomatic index was calculated using adjusted body mass (i.e., total body mass minus liver mass) to reduce mathematical coupling between the numerator and denominator. By using adjusted body mass, we aimed to improve statistical independence of the variables and provide a more accurate representation of treatment effects on liver investment relative to the rest of the body. Finally, we used a dissecting microscope (ZEISS Stemi 508) to inspect the gross morphology of the gonads and determine the sex of the metamorph.

Blood smear processing and cell identification was completed as described above in the week 2 endpoints section, and following Gavel et al. (2019). For BKA assays, we adapted the methods used by Venesky et al. (2012) and Gomes et al. (2012). We conducted five separate assays, using the plasma of one tadpole per mesocosm during each assay. Each assay used plasma from a new tadpole, and fresh stock solutions were prepared for each assay. On the day of the assay, we re‐constituted an *Escherichia coli* pellet (ATCC 8739; Microbiologics, USA) according to manufacturer instructions. The pellets were then diluted in phosphate buffered saline (PBS; Gibco lot 2522980) to a working concentration of ~36,000 colony‐forming units (CFU) per 1 mL to create an *E. coli* stock solution. For each sample, we combined and vortexed 10 µL of plasma with 20 µL *E. coli* stock solution and 50 µL PBS. We allowed the challenge to proceed at room temperature for 30 min and then added another 100 µL of PBS to each sample. We kept stock solutions in the fridge when not in use and kept samples over ice after adding the additional 100 µL of PBS, until the sample was ready to be plated. We further prepared three control replicates for each assay, prepared the same way as above with 10 µL of PBS substituted for plasma. The controls were prepared in triplicate at the start, middle and end of sample preparation.

For each sample and control, we vortexed before plating 50 µL on a plate of nutrient agar in triplicate (Formulation: ATTC Medium 3, using BP1423‐500 Agar and Difco Nutrient Broth). We then incubated (Forma Steri‐Cycle CO2 Incubator, Model 370) the plates at 37°C for 18 h. Following incubation, colonies on each plate were counted. Bacteria killing ability was calculated as:

$$\frac{\left({\mathrm{Average colonies}}_{\mathrm{Control}}-{\mathrm{Average colonies}}_{\mathrm{Sample}}\right)}{{\mathrm{Average colonies}}_{\mathrm{Control}}}\times 100$$

### 2.8 Statistical Analyses

To assess differences in non‐count response variables, we used general linear mixed models (GLMM) in R studio version 2022.12.0.353 using R version 4.2.2 (R Core Team 2022) using Gaussian distributions and a log link function using the lme4 package and lmerTest (Kuznetsova et al. 2017). GLMMs allow for the inclusion of random effects, such as the non‐independence of tadpoles/metamorphs from the same tank (Zuur et al. 2009). The fixed effects for this initial model included temperature (warmed vs. ambient), herbicide (control vs. atrazine), as well as their interaction. We also included biologically relevant traits such as body mass, SVL and sex to account for their potential influence on response variables. We included random variables in our models including block (to account for any added variation based on position of the mesocosm in the research garden), tank (to account for non‐independence of tadpoles reared in the same mesocosm), date (to account for differences in temperature on different days—that is, it got warmer as it moved into summer) and assay number (to account for non‐independence of BKA samples assayed at the same time). When including both block and tank in models, we nested tank within block. When random variables did not add any variation (singular fit), the random variables were removed, and general linear models were run instead.

We used Akaike Information Criterion comparisons (AIC; Supporting Information S1: Tables 2, 3; Nakagawa and Cuthill 2007) and backwards selection to select best‐fit models. For initial count models (traits) we used generalized linear mixed models with Poisson distributions using the glmmPQL package, to avoid overdispersion (Venables and Ripley 2002). For binary data (e.g., sex, survival), we used a binomial distribution. We then used the emmeans package (Russell et al. 2024) to run pairwise comparisons between individual treatments while accounting for random effects.

When running tadpole leukocyte ratio models (neutrophil/lymphocyte, eosinophil/leukocyte, monocyte/leukocyte), we removed tadpoles with < 20 leukocytes counted, to avoid artificially inflated ratios caused by low leukocyte numbers.

We sampled the first 20 individuals to complete metamorphosis from tanks (total of *N* = 360). These, as well as the remaining frogs were included in the analyses of survival (*N* = 717), as well as completion of metamorphosis (*N* = 717). Three metamorphs were caught in between the pond liners and mesocosms and subsequently died. These individuals were excluded from all metamorph analyses. Due to initial difficulties collecting blood for BKA assays, we sampled blood from five additional metamorphs (i.e., the 21st to 25th metamorphs to complete metamorphosis) in warmed treatments.

For mean distance jumped, only metamorphs that were able to complete the required 11 jumps were included. We further did not include jumps made by metamorphs to reach the side of the arena, as these jumps were typically shorter, and likely representative of shelter seeking. For mean prods required to jump and mean distance jumped, we did not include measures when the metamorph reached the end of the runway as these measures were typically shorter, given the metamorph had reached the grass patch.

We followed Zuur et al. (2009) and Gavel et al. (2019) to complete model selection and validation. When models did not meet assumptions for parametric models, we ran non‐parametric Kruskal‐Wallis tests, followed by Dunn's tests to assess whether there were any differences in measured traits.

Two tanks (Ambient control, replicate two, and ambient atrazine, replicate five) were removed from all statistical analyses due to occurrences of abdominal edema that can be suggestive of bacterial infection or other fluid balance disorders (Pessier 2009).

## 3 Results

### 3.1 Atrazine Concentrations and Temperature

Measured concentrations of atrazine in herbicide‐treated mesocosms were 2.7 ± 0.20 µg/L at the start of the experimental period (May 17, 2023), and 1.11 ± 0.16 µg/L at the end of the experimental period (July 17, 2023), indicating a breakdown of 41% over 61 days (Supporting Information S1: Table 4). Very slight atrazine contamination was detected in control mesocosms (atrazine concentrations in controls ranged from not detected (i.e., below method detection limit of 0.0005 µg/L) to 0.002 µg/L, likely due to the atrazine that entered the mesocosms from rainfall (atrazine in rainfall ranged from 0.010 µg/L to 0.128 µg/L). Temperatures in the ambient treatment ranged from 7.18°C to 29.55°C over the experimental period, whereas temperatures in the warmed treatment ranged from 10.16°C to 31.27°C over the experimental period (Supporting Information S1: Figure 6). Temperature difference between ambient and warmed tanks averaged 2.30 ± 0.73°C over the course of the experimental period (Supporting Information S1: Figure 6). Temperatures in warmed mesocosms were significantly higher than temperatures in the ambient temperature mesocosms (Supporting Information S1: Table 5).

### 3.2 Tadpole Endpoints

We sampled 378 tadpoles for apical endpoints after 2 weeks of exposure to their respective treatments. We found no significant differences in tadpole survival over the first 2 weeks of the experimental period (Table 1, Supporting Information S1: Table 6). For stage of development, our initial model indicated that tadpoles in warmed tanks exhibited significantly accelerated development compared to those in ambient tanks (Table 1, Supporting Information S1: Tables 7, 8; Figure 1). We further found there was a significant negative interaction between atrazine and temperature. Specifically, while temperature increased development, atrazine decreased it (Supporting Information S1: Table 7). When parsed by individual treatment, we found that warmed control tadpoles were significantly more developed than either ambient treatment (Supporting Information S1:Table 8; Figure 1). Tadpole SVL was significantly increased in warmed treatments compared to ambient ones (Table 1, Supporting Information S1: Tables 7, 8; Figure 1). Specifically, when parsed by treatment, tadpoles in the warmed control treatment were significantly larger than tadpoles from either ambient treatment (regardless of atrazine treatment; Supporting Information S1: Table 8; Figure 1). Similarly, tadpoles in the warmed atrazine group were significantly larger than those in the ambient control group (Supporting Information S1: Table 8; Figure 1).

**Table 1:** Mean (±standard deviation) of endpoints collected from *Lithobates pipiens* tadpoles after 2 weeks of exposure to either atrazine (2 μg/L) or dechloraminated water that was either warmed (Warmed Control, Warmed Atrazine) or ambient (Ambient Control, Ambient Atrazine).

	Ambient control	N	Warmed control	N	Ambient atrazine	N	Warmed atrazine	N
SVL	11.8 (1.8)	83	14.1 (2.5)	105	11.8 (1.7)	84	13.2 (2.1)	105
Stage	26 (1)	83	28 (1)	105	27 (1)	84	27 (1)	105
Survival	100 (0)	244	100 (0)	305	100 (0)	244	99 (1)	305
Proportion Startled^a^	0.97 (0.06)	32	0.95 (0.07)	40	0.94 (0.07)	32	0.93 (0.07)	40
Active intervals pre^b^	18 (11)	32	10 (10)	40	21 (8)	32	13 (10)	40
Active intervals post^b^	17 (8)	32	13 (7)	40	20 (6)	32	14 (8)	40
Erythrocytes^c^	1564 (852)	30	2311 (1216)	32	1717 (906)	28	1926 (1099)	35
Leukocytes^c^	46 (34)	30	92 (62)	32	50 (32)	28	80 (67)	35
Leukocyte/Erythrocyte^c^	0.03 (0.02)	30	0.04 (0.02)	32	0.03 (0.02)	28	0.04 (0.02)	35
Eosinophil/Leukocyte^c^	0.04 (0.05)	26	0.05 (0.04)	29	0.06 (0.07)	23	0.05 (0.04)	32
Monocyte/Leukocyte^c^	0.00 (0.00)	26	0.00 (0.00)	29	0.00 (0.00)	23	0.00 (0.00)	32
N/L^c^	0.14 (0.12)	26	0.07 (0.08)	29	0.21 (0.16)	23	0.12 (0.16)	32

*Note*: Endpoints include snout to vent length (SVL; mm), stage of development (Stage), mean survival (%) to experimental day 16 (June 2, 2023), startle response, time active before startle (active intervals pre), time active post startle (active intervals post), erythrocyte and leukocyte counts, leukocyte to erythrocyte ratios, monocyte to leukocyte ratios, eosinophil to leukocyte ratios, and neutrophil to lymphocyte ratios. Blood smear counts are based on the enumeration of 100 fields of view for each sample, and for leukocyte differentials (eosinophil/leukocyte, monocyte/leukocyte, and neutrophil/lymphocyte (N/L) ratios); proportions only include individuals who had at least 20 leukocytes counted. Sample size included as N. For significance levels for initial and parsed models, see Figure 1, Supporting Information S1: Tables 6, 8, 9, 13, or main text.

^a^ Startle response presented as the proportion of tadpoles in each treatment that displayed a startle response.

^b^ Time active before startle, and time active post startle presented as mean (+/− standard deviation) of time across 10‐s intervals that a tadpole was active for a period of 5 min (for a total of 30 intervals), before and after startle.

^c^ Counts are based on the enumeration of 100 fields of view for each sample, whereas proportions only include individuals who had at least 20 leukocytes counted.

For locomotor performance trial analyses, we assessed 44 tadpoles. We found no significant differences in the proportion of tadpoles startled by the bar dropping mechanism (Supporting Information S1: Table 6). In fact, startle responses were very high across all treatments (range 93%–97% of tadpoles startled; Table 1), indicating the bar dropping mechanism was an effective startle mechanism. For locomotor activity, we found significant differences between treatments, both before and after the startle (Table 1, Supporting Information S1: Table 6; Figure 1). Before the startle, tadpoles in warmed treatments (warmed control, warmed atrazine) were significantly less active than tadpoles in either ambient treatment (ambient control, ambient atrazine). However, after the startle, tadpoles in warmed treatments (warmed control, warmed atrazine) were significantly less active than only tadpoles in the ambient atrazine treatment (Table 1, Supporting Information S1: Table 6; Figure 1).

We were able to obtain 125 usable blood smears from tadpoles. Some smears had to be excluded due to poor quality (too sparse/too dense). However, for leukocyte differential analyses, after removing tadpoles with < 20 leukocytes observed, we were left with 110 tadpoles sampled. Our initial models found no significant differences in total erythrocyte counts (Table 1, Supporting Information S1: Tables 7, 8), but did find that tadpoles in the warmed treatments had significantly higher total leukocyte counts, compared to ambient treatment tadpoles (but no effects of atrazine; Table 1, Supporting Information S1: Table 7); however, these results were no longer significant when parsed by individual treatment (Supporting Information S1: Table 8). We further found no significant differences in leukocyte/erythrocyte ratios between treatments (Supporting Information S1: Table 6). However, we did find significant differences in NL ratios between treatments (Table 1, Supporting Information S1: Table 6; Figure 1). Specifically, we found that tadpoles in both ambient treatments (ambient control, ambient atrazine) had significantly higher NL ratios compared to tadpoles in the warmed control group (Supporting Information S1: Table 6; Figure 1). Similarly, tadpoles in the ambient atrazine treatments had significantly higher NL ratios compared to warmed atrazine tadpoles (Supporting Information S1: Table 6; Figure 1). There were no significant differences in eosinophil to leukocyte ratios or monocyte to leukocyte ratios between treatments (Table 1, Supporting Information S1: Table 6).

### 3.3 Metamorph Endpoints

Our initial model indicated there was significantly lower survival in the warmed treatments compared to ambient treatments (but no effect of atrazine; Table 2, Supporting Information S1: Table 5). However, when parsed by individual treatment, this relationship was no longer significant (Supporting Information S1: Table 9). Our initial model indicated that temperature, but not atrazine, had a significant effect on days to metamorphosis (Table 2, Supporting Information S1: Table 5). Specifically, when parsed by individual treatment, tadpoles in warmed tanks completed metamorphosis in significantly less time (7–8 days earlier, on average) compared to those in ambient treatments (Table 2, Supporting Information S1: Table 9; Figure 2). We found no significant differences in the overall number of metamorphs to complete metamorphosis by the end of the experimental period, sex or hepatosomatic index between each treatment (Table 2, Supporting Information S1: Tables 5, 9, 10).

**Table 2:** Mean (±standard deviation) of endpoints collected from *Lithobates pipiens* metamorphs after exposure to either atrazine (2 μg/L) or dechloraminated water that was either warmed (Warmed Control, Warmed Atrazine) or ambient (Ambient Control, Ambient Atrazine).

Ambient control	N	Warmed control	N	Ambient atrazine	N		Warmed atrazine	N
Survival	98.88 (2.39)	160	91.39 (5.79)	198	97.5 (3.53)	160	95.47 (2.75)	199
Body Mass	3.16 (0.43)	80	2.62 (0.43)	99	3.09 (0.44)	81	2.55 (0.42)	100
SVL	36.85 (2.40)	80	34.14 (1.80)	99	36.52 (2.19)	81	33.79 (2.09)	100
Femur Length	13.93 (12.13)	80	0.90 (0.85)	99	13.62 (0.82)	81	12.08 (0.85)	100
Tibiofibular Length	14.58 (1.26)	80	13.45 (0.87)	99	14.35 (1.10)	81	13.28 (0.97)	100
Days to Metamorphosis	57.94 (2.35)	157	49.71 (2.95)	189	57.11 (2.00)	160	49.66 (2.80)	192
Hepatosomatic Index	2.83 (0.42)	40	2.93 (0.59)	50	2.83 (0.43)	39	2.83 (0.58)	50
Mean Distance Jumped	0.27 (0.07)	34	0.28 (0.08)	40	0.27 (0.05)	30	0.27 (0.07)	35
Prods Locomotor	0.24 (0.52)	40	0.23 (0.38)	48	0.23 (0.41)	40	0.38 (0.68)	47
Pass % Locomotor	87.50 (5.00)	40	85.33 (12.61)	48	77.50 (5.00)	40	73.56 (14.98)	47
Max Distance Jumped	0.39 (0.11)	40	0.39 (0.14)	48	0.38 (0.09)	40	0.38 (0.14)	47
% Jumped to End	9.03 (11.87)	40	11.07 (18.57)	48	12.50 (14.43)	40	10.00 (13.69)	47
BKA	41.23 (16.75)	15	33.13 (17.35)	13	41.72 (20.00)	12	34.61 (19.12)	9
Erythrocytes	4477 (1762)	19	4439 (1482)	25	4638 (1370)	19	4273 (1484)	25
Leukocytes	153 (89)	19	110 (47)	25	133 (58)	19	117 (65)	25
Leukocyte/Erythrocyte	0.04 (0.02)	19	0.03 (0.01)	25	0.03 (0.01)	19	0.03 (0.01)	25
Monocyte/Leukocyte	0.01 (0.02)	19	0.03 (0.02)	25	0.02 (0.02)	19	0.02 (0.02)	25
Eosinophil/Leukocyte	0.02 (0.01)	19	0.03 (0.03)	25	0.02 (0.02)	19	0.02 (0.03)	25
N/L	0.06 (0.03)	19	0.05 (0.03)	25	0.05 (0.03)	19	0.03 (0.02)	25
Blood Glucose	1.72 (0.61)	35	2.28 (0.82)	46	1.93 (0.90)	38	2.43 (0.86)	47

*Note*: Endpoints include survival (%), body mass (g), snout to vent length (SVL; mm), femur length (mm), tibiofibular length (mm), days to metamorphosis, hepatosomatic index (%), mean distance jumped (m), mean prods required to jump (max three), pass or fail rate for locomotor assay (% passed), maximum distance jumped (m), percent of frogs that jumped to the end of the locomotor runway (%), bacteria killing ability (BKA; % bacteria killed), total erythrocytes, total leukocytes, leukocyte to erythrocyte ratio, monocyte to leukocyte ratio, eosinophil to leukocyte ratio, neutrophil to lymphocyte (N/L) ratio, blood glucose (mmol/L). Sample size included as N. For significance levels for initial and parsed models, see Figure 2, Supporting Information S1: Tables 6, 9, 10, or main text.

Body mass and SVL were significantly affected by temperature, but not atrazine (Table 2, Supporting Information S1: Tables 5, 9). Namely, both initial and parsed models indicated that metamorphs in warmed treatments were smaller and weighed less compared to those in ambient treatments (Table 2, Supporting Information S1: Tables 5, 9; Figure 2). When accounting for SVL we found no difference in relative tibiofibular length (Table 2, Supporting Information S1: Tables 5, 9). Contrastingly, both initial and parsed models indicated that femur length was shorter in warmed treatments, even when accounting for differences in SVL in the model (Table 2, Supporting Information S1: Tables 5, 9). Of the 180 metamorphs assessed for their locomotor performance, we were able to collect data from 175 individuals. Despite the differences in femur length, we found no differences in mean distance jumped, maximum distance jumped, or likelihood to jump to the end of the runway between treatments in either our initial or parsed models (Table 2, Supporting Information S1: Tables 5, 9, 10). Similarly, we found no significant differences in mean prods required to jump, or pass/fail rates between treatments for the locomotor performance assay in either our initial or parsed models (Table 2, Supporting Information S1: Tables 5, 9, 10).

Glucose values were obtained from 167 metamorphs (some samples gave error codes and could not be used). We found significant differences in blood glucose levels between ambient and warmed treatments (Table 2, Supporting Information S1: Tables 5, 9; Figure 2). Specifically, blood glucose levels were significantly elevated in both warmed treatments (warmed control, warmed atrazine) compared to the ambient controls. Similarly, glucose levels were significantly elevated in warmed atrazine metamorphs compared to ambient atrazine metamorphs (Table 2, Supporting Information S1: Tables 5, 9; Figure 2). We sampled 90 metamorphs for BKA assays, and of those, we were able to collect BKA data from 49 samples. Many samples were excluded due to the low blood volume collected. We found no significant differences in BKA between treatments in either our initial or parsed models (Table 2, Supporting Information S1: Tables 5, 9).

For blood cell profiles, we quantified a total of 88 blood smears (Table 2). We found no significant differences in total erythrocyte or leukocyte counts between treatments, either in our initial or parsed models (Supporting Information S1: Tables 5, 9). Our initial model indicated that atrazine had no significant effect on leukocyte to erythrocyte ratios, but did find that these ratios were significantly lower in warmed treatments compared to ambient ones (Supporting Information S1: Table 5). However, when parsed by treatment, this relationship was no longer significant (Supporting Information S1: Table 9). NL ratios were significantly lower in warmed atrazine metamorphs compared to both ambient and warmed control metamorphs (Table 2, Supporting Information S1: Table 10; Figure 2). We also found that monocyte to leukocyte ratios were significantly elevated in warmed controls compared to ambient controls but found no significant effects of atrazine (Table 2, Supporting Information S1: Table 10; Figure 2). Contrastingly, we found no significant differences in eosinophil to leukocyte ratios between any of the treatments (Table 2, Supporting Information S1: Table 10).

### 3.4 Physicochemical Water Quality Variables

We monitored a suite of physicochemical water quality variables throughout the exposure and found they were within acceptable guideline ranges (Supporting Information S1: Table 11; Canadian Council on Animal Care CCAC 2021b; OECD 2009, 2015) and were similar to other mesocosm experiments (Robinson et al. 2017, 2019). Specifically, DO ranged from 56% to 106%, pH ranged from 6.72 to 7.46, conductivity ranged from 216.9 to 633.0 µS/cm, unionized ammonia ranged from 0 to 0.10 mg/L, nitrate ranged from 0 to 5.0 ppm, nitrite ranged from 0 to 0.50 ppm, hardness ranged from 46.5 to 107.4 ppm, and water temperature ranged from 7.18°C to 31.27°C.

## 4 Discussion

Our study examined the individual and combined effects of atrazine and warming on *L. pipiens* at two developmental stages. We found that temperature alone had significant effects on both tadpoles and metamorphs, whereas low concentrations of atrazine alone did not. However, when combined with elevated temperatures, atrazine significantly affected tadpole development and metamorph NL ratios. Given the scope of the study, we focus our discussion on significant and salient results keeping in mind that elevated temperatures with atrazine at low to moderate concentrations is a likely future scenario for amphibian habitats in and around the Great Lakes region.

### 4.1 Effects of Temperature—Survival

While warming did not affect tadpole survival initially, by the end of the experiment, survival was significantly lower in warmed treatments compared to ambient treatments. Our results contrast a previous study using another ranid species (Table 3). The water temperatures during the first 2 weeks of this study were well within the tolerable range for *L. pipiens* (Green and Taylor 2009), which may explain the initially high survival rates.

**Table 3:** Effects of atrazine and/or temperature (Temp) on endpoints measured in this study on amphibians cited in the discussion.

Development stage	Endpoint	Treatment	Concentration (μg/L)/Temperature (°C)	Species	Effect (ref)	Effect (this paper)	Reference
Tadpole	Survival	Atrazine	20, 200, 2000, 20 000	*Rana pipiens, Rana sylvatica, Anaxyrus americanus*	—	—	Allran and Karasov (2001)
			0.18, 1.8, 18, 180	*A. americanus*	—		Paetow et al. (2023)^a^
		Temp	17, 22, **27**	*Rana clamitans*	↓	—	Boone and Bridges (1999)^a^
	Gosner stage	Atrazine	**1**, 25	*Xenopus laevis*	↓	—	Floyd et al. (2008)
			1, 25	*R. sylvatica*	—		
			**0.18**, **1.8**, 18, **180**	*A. americanus*	↓		Paetow et al. (2023)^a^
		Temp	23, **28**	*R. pipiens*	↑	↑	Freitas et al. (2017)^a^
	Size	Atrazine	0.18, **1.8**, 18, **180**	*A. americanus*	↓	—	Paetow et al. (2023)^a^
		Temp	18, **22**	*Limnodynastes peronii*	↑	↑	Courtney Jones et al. (2015)
			15, 21, 27	*Quasipaa boulengeri*	—		Fan et al. (2021)^a^
			23, **28**	*R. pipiens*	↑		Freitas et al. (2017)^a^
			29, **32**, **34**	*Polypedates cruciger*	↓		Weerathunga and Rajapaksa (2020)^a^
	Eosinophils	Atrazine	**3**, **30**	*R. sylvatica*	↓	—	Blaustein et al. (2004)^b^
		Temp	21, **25**, **29**	*Pelobates cultripes*	↓	—	Burraco and Gomez‐Mestre (2016)
	NL^f^ ratio	Temp	21, **25**, **29**	*P. cultripes*	↓	↓	
	Swimming speed	Atrazine	20, 200, 2000, 20 000	*R. pipiens, R. sylvatica, A. americanus*	—	—	Allran and Karasov (2001)
	Locomotor activity	Atrazine	**178**	*Osteopilus septentrionalis*	↑	—	Ehrsam et al. (2016)
			**0.1**, **1**, **10**, **100**	*Euphlyctis cyanophyctis*	↑		Supekar and Gramapurohit (2023)
		Temp	29, **32**, **34**	*P. cruciger*	↓	↓	Weerathunga and Rajapaksa (2020)
Metamorph	Survival	Atrazine	0.1, 1, 10, 100, **1000**	*Rhinella arenarum*	↓	—	Brodeur et al. (2013)
			20, 200, 2000	*Hyla versicolor*	—		Diana et al. (2000)
			0.1	*R. pipiens*	—		Hayes et al. (2006)
			0.1, **1.8**	*R. pipiens*	↓		Langlois et al. (2010)^c^
			4, 40, 400	*Ambystoma barbouri*	‐/↓^d^		Rohr et al. (2011)^e^
		Temp	25, 30	*Bufo terrestris*	—	↓	Beck and Congdon (2000)
			18, **22**, **26**	*L. peronii*	↓		Courtney Jones et al. (2015)^a^ ^c^
			29, **32**, **34**	*P. cruciger*	↓		Weerathunga and Rajapaksa (2020)
			16, 19, 22, 25	*A. barbouri*	—		Rohr et al. (2011)
		Atrazine x Temp	4, 40, 400 (A), 16, 19, 22, 25 (T)	*A. barbouri*	—	—	
	Development rate	Atrazine	0.1, **1**, **10**, **100**, 1000	*R. arenarum*	↑	—	Brodeur et al. (2013)
			20, 200, 2000	*H. versicolor*	—		Diana et al. (2000)
			0.1	*R. pipiens*	—		Hayes et al. (2006)
			0.1, 1, 10, **100**	*E. cyanophlyctis*	↑		Supekar and Gramapurohit (2023)^c^
			**4**, **40**, **400**	*A. barbouri*	↓		Rohr et al. (2011)^e^
		Temp	25, **30**	*B. terrestris*	↑	↑	Beck and Congdon (2000)
			18, 22, 26	*L. peronii*	—		Courtney Jones et al. (2015)^a^^,^ c
			23, **28**	*R. pipiens*	↑		Freitas et al. (2017)^a^
			29, **32**	*P. cruciger*	↓		Weerathunga and Rajapaksa (2020)
			16, **19, 22, 25**	*A. barbouri*	↑		Rohr et al. (2011)^e^
		Atrazine x Temp	**4**, **40**, **400** (A), 16, **19**, **22**, **25** (T)	*A. barbouri*	↑	—	
	Body mass	Atrazine	0.1, **1**, **10**, 100, 1000	*R. arenarum*	↑	—	Brodeur et al. (2013)
			20, **200**, **2000**	*H. versicolor*	↓		Diana et al. (2000)
			**0.1**	*R. pipiens*	↓		Hayes et al. (2006)
			0.1, 1, 10, 100	*E. cyanophlyctis*	—		Supekar and Gramapurohit (2023)^a^^,^ c
			0.1, 1.8	*R. pipiens*	—		Langlois et al. (2010)^c^
			**4**, **40**, **400**	*A. barbouri*	↓		Rohr et al. (2011)^e^
		Temp	25, **30**	*B. terrestris*	↓	↓	Beck and Congdon (2000)
			16, 19, 22, 25	*A. barbouri*	—		Rohr et al. (2011)^e^
		Atrazine x Temp	**4**, **40**, **400** (A), 16, **19**, **22**, **25** (T)	*A. barbouri*	↑	—	
	Size	Atrazine	0.1, **1**, **10**, 100, 1000	*R. arenarum*	↑	—	Brodeur et al. (2013)
			20, **200**, **2000**	*H. versicolor*	↓		Diana et al. (2000)
			**0.1**	*R. pipiens*	↓		Hayes et al. (2006)
			0.1, 1.8	*R. pipiens*	—		Langlois et al. (2010)^c^
			0.1, 1, 10, 100	*E. cyanophlyctis*	—		Supekar and Gramapurohit (2023)^a^^,^ c
		Temp	18, 22, 26	*L. peronii*	—	↓	Courtney Jones et al. (2015)^a^
	Proportion female	Atrazine	0.1, **1.8**	*R. pipiens*	↑	—	Langlois et al. (2010)^c^
	Completion of metamorphosis	Atrazine	0.1, **1.8**	*R. pipiens*	↓	—	
	Sprint speed	Temp	25, 30	*B. terrestris*	—	—	Beck and Congdon (2000)
	NL^f^	Temp	29, **32**	*P. cruciger*	↑	—	Weerathunga and Rajapaksa (2020)
	Eosinophils	Temp	29, 32	*P. cruciger*	—	—	
	Monocytes	Temp	29, **32**	*P. cruciger*	↑	↑	
	WBC/RBC^f^	Temp	29, **32**	*P. cruciger*	↓	↓	

*Note*: Effect is compared back to control for atrazine treatments, or cooler temperature for temperature treatments. Effect (ref) refers to the cited paper, whereas effect (this paper) refers to effects observed in the present study. Arrows pointing up indicate an increase in the measured trait, arrows pointed down indicate a decrease in the measured trait, and horizontal lines indicate no significant effect. Bolded concentrations/temperatures are significant at *p* < 0.05 according to the published results.

^a^ Endpoint sampled at multiple intervals; comparisons were selected based on the exposure duration that most closely reflected the exposure duration used in this study (closest to 2 weeks of exposure for tadpoles, and closest to the completion of metamorphosis for metamorphs).

^b^ Tadpoles exposed to both atrazine and parasites.

^c^ Endpoint sampled at metamorphic climax (Gosner stage 42).

^d^ Significance varied depending on statistical testing used.

^e^ Paper used a regression‐based approach across different temperatures/concentrations of atrazine.

^f^ Acronyms used: NL = Neutrophil/Lymphocyte ratio, WBC = Leukocytes, RBC = Erythrocytes.

Temperature can have a strong effect on amphibian mortality. For example, *P. cruciger* reared at temperatures only 3°C higher than ambient controls experienced 100% mortality, shortly following metamorphosis (Weerathunga and Rajapaksa 2020). While we did not maintain froglets following metamorphosis, we note that three metamorphs in the warmed control treatment died shortly following their locomotor performance assessment, before euthanasia. Future studies should investigate the carry‐over effects of rearing *L. pipiens* at warmer temperatures.

### 4.2 Effects of Temperature—Development

Warmer temperatures accelerated growth and development in tadpoles, consistent with previous findings on developmental plasticity in *L. pipiens* (Brannelly et al. 2019; Freitas et al. 2017). At the metamorph stage, warmed treatment individuals completed metamorphosis significantly faster than those in ambient treatments, consistent with findings from previous studies (Table 3). Accelerated development is important not only to avoid possible desiccation but can also help amphibians avoid prolonged exposure to aquatic contaminants such as pesticides (Rohr et al. 2011).

In addition to a more rapid development, metamorphs from warmed treatments were significantly smaller than ambient treatment individuals, highlighting a trade‐off between development speed and size (Ruthsatz et al. 2018). Size at metamorphosis can be affected by factors such as density, food availability, and water depth (Courtney Jones et al. 2015; Ding et al. 2015; Kohli et al. 2019). However, the tadpoles in this study were kept at low densities, with ample food, and water levels were topped up to avoid possible effects of hydroperiod. Thus, the observed effects are likely the result of temperature.

Warmer temperatures (within an optimal thermal window) can accelerate both growth rates and differentiation rates (Smith‐Gill and Berven 1979), however, when faced with stressful conditions, energy can be preferentially diverted into differentiation, resulting in less growth (Richter‐Boix et al. 2011). Under stressful environmental conditions, thyroid hormones can synergize with stress hormones to accelerate development (Bonett et al. 2010; Paul et al. 2022). Interestingly, blood glucose was also significantly higher in warmed treatment metamorphs. Blood glucose can rise in response to stress but may also result from a temperature‐induced increase in metabolic rate, as higher temperatures can elevate metabolism in ectotherms (Rollins‐Smith and Le Sage 2023). As many amphibians, including *L. pipiens*, do not eat while completing metamorphosis, the blood glucose levels represent fasting levels, and were not influenced by potential food intake.

These developmental shifts may compromise other functions important for survival. Whereas larger tadpoles generally experience less predation (Brodie and Formanowicz 1983), accelerated development may reduce investment into traits like locomotor performance, or cellular immunity, thus increasing vulnerability in terrestrial stage amphibians (Altwegg and Reyer 2003; Gervasi and Foufopoulos 2008).

### 4.3 Effects of Temperature—Locomotor Performance

Warmed treatment tadpoles showed reduced activity, compared to those from ambient treatments. This response has also been noted in *P. cruciger* (Table 3). Contrastingly, there were no significant differences in locomotor performance at the metamorph stage, despite the smaller body size and shorter femur lengths of metamorphs from warmed treatments. These results were surprising, given that previous research found shorter development times can affect metamorphic locomotor performance, with larger individuals outperforming their smaller counterparts (Goater et al. 1993; Beck and Congdon 2000).

While the effect of temperature on tadpole locomotor performance was temporary, these findings are concerning. Lower activity may place tadpoles at greater risk for predation and parasitism (Koprivnikar et al. 2006). Given that parasites are predicted to acclimate to temperature shifts more quickly than their hosts (Cohen et al. 2017), the potential trade‐offs between energy for growth and its effects on anti‐parasite efficacy should be investigated.

### 4.4 Effects of Temperature—Stress and Immunity

Elevated NL ratios (two to three times higher) in ambient treatment tadpoles, compared to warmed treatment tadpoles may reflect a moderate stress response (Table 1; Davis and Maerz 2011). Similar responses have also been found in non‐ranid species (Table 3). Individuals can experience physiological stress when faced with temperatures outside of their thermal window (Ruthsatz et al. 2018), which in turn can suppress aspects of immune function. Chronically elevated NL ratios have been associated with reduced immunocompetence (Dhabhar 2002), suggesting that ambient‐treatment tadpoles may have been more vulnerable to infection than those in warmed treatments. The degree at which they are vulnerable will also depend on the temperature optimum for parasite infectivity.

At metamorphosis, individuals in the warmed treatments had lower leukocyte to erythrocyte ratios, compared to ambient treatment individuals. Our results align with a previous study on a non‐ranid species (Table 3). While studies investigating the effects of warmer temperatures on blood cells in recently metamorphosed amphibians are sparse, previous studies that investigated the effects of reduced hydroperiod on amphibian immune function can provide additional insight (Rollins‐Smith and Le Sage 2023). For example, *R. sylvatica* reared under desiccation regimes also had lower leukocytes compared to frogs reared at consistent water levels (Gervasi and Foufopoulos 2008). Warmer temperatures can accelerate amphibian development; however, this can come at the cost of reduced immunocompetency and increased stress (reviewed by Kohli et al. 2019). This reduction in immunocompetency is likely the result of an exchange between energy for growth versus energy for immune function (Warne et al. 2011).

Interestingly, metamorphs in the warmed control treatment had significantly elevated proportions of monocytes, compared to ambient control metamorphs. Increased monocytes in response to warming have also been shown in a non‐ranid amphibian (Table 3). Similar responses have also been observed in fish reared at warmer temperatures (Dinken et al. 2022), and in fish exposed to heat waves (Dittmar et al. 2014). In our study, many of the warmed treatment individuals completed metamorphosis approximately a week before ambient treatment individuals (Table 1). Notably, the completion of metamorphosis of warmed treatment individuals (mean of 49 days), corresponded with a heat wave during our study season (Supporting Information S1: Figure 6). Given monocytes play an important role in phagocytosis and inflammation (Turner 1988), further research should examine whether proliferation of monocytes is a response of *L. pipiens* to increased temperatures.

Despite the effects of warming temperatures on leukocyte differentials and blood glucose, we found no differences in plasma BKA between treatments. Our results contrast previous studies which found that elevated temperatures and shorter hydroperiods can reduce BKA in amphibians (Brannelly et al. 2019; Lima et al. 2020). Variations in methodology and the magnitude of temperature differentials may explain these discrepancies.

Given the role of emerging diseases in amphibian declines, it is concerning that an increase in mean temperature of only 2.3°C can negatively affect immunity in *L. pipiens* metamorphs. Recently metamorphosed amphibians are immunologically vulnerable, and can be especially susceptible to ranaviruses (Rollins‐Smith 2017; Rollins‐Smith 1998; Green et al. 2002), emerging pathogens implicated in global amphibian mass mortality events (Green et al. 2002; Haislip et al. 2011; Miller et al. 2011). Given warming temperatures can further reduce metamorph immune function, while concurrently increasing the pathogenicity of ranaviruses (Brand et al. 2016; Hall et al. 2018), warming may increase *L. pipiens'* susceptibility to infection.

### 4.5 Effects of Atrazine

We found no significant effects of atrazine, individually, on any metrics at either life history stage. Previous studies have found that amphibians exposed to similar concentrations of atrazine showed significant differences in metrics assessed herein (Table 3), highlighting the species‐ or study‐ specific nature of amphibian responses to contaminants.

The high survival in metamorphs exposed to atrazine was unexpected, given a previous study found that *R. pipiens* exposed to a similar concentration of atrazine (1.8 μg/L) from stage 25 to metamorphic climax had significantly lower survival compared to controls (66% and 79%; respectively; Langlois et al. 2010). This discrepancy may be due to a husbandry effect, as our ambient controls had higher survival than those in Langlois et al. (99% vs. 79%, respectively). Furthermore, while Langlois et al. (2010) and our study both used outdoor mesocosms, our tanks were buried in the ground, to reduce daily temperature fluctuations, which can magnify the toxicity of pesticides (Verheyen et al. 2022).

### 4.6 Effects of Temperature x Atrazine

Temperature and atrazine can have opposing effects on tadpole development, with previous studies showing that atrazine can slow the rate of development, while warmer temperatures can increase it (Table 3). These conflicting effects are evidenced by the significant negative interaction between atrazine and temperature in stage of development (Supporting Information S1: Table 7). While we did not find any significant differences in stage between atrazine treatments at ambient temperatures, we note that at warmer temperatures, tadpoles in the control treatment tended to be more developed than those in the atrazine treatment (*p* = 0.05; Supporting Information S1: Table 7). Notably, the interaction was no longer evident at the metamorph stage. Thus, at the tadpole stage, it seems warming temperatures may temporarily offset an atrazine‐induced delay in development in *L. pipiens*.

We also found that atrazine and temperature can combine to affect stress and/or immunocompetence in recently metamorphosed individuals, as evidenced by NL ratios. Whereas elevated NL ratios represent a mild to moderately stressed individual, under severe stress, NL ratios can become depleted (Maxwell 1993; Davis et al. 2008). Metamorphs in the warmed atrazine treatment had significantly lower NL ratios than either control treatment, regardless of temperature. This decrease may be indicative of a synergistic effect, wherein atrazine and warmed temperatures combined to have exacerbated effects, thus further compromising immunity. Both accelerated metamorphosis and detoxification can be energetically costly, and these processes may have come at the cost of reduced immune function. Decreases in NL ratios in response to pesticide exposure in amphibians have previously been documented (Gavel et al. 2021; Lajmanovich et al. 2015). Previous research has found that independently, warmed temperatures can increase circulating neutrophils in amphibians (Weerathunga and Rajapaksa 2020). However, the effects of atrazine on amphibian NL ratios are varied. While one study found that exposure to atrazine (2.1 μg/L) post‐metamorphosis had no effect on NL ratios in *L. pipiens* (Paetow et al. 2012), another study found that adult *R. pipiens* exposed to atrazine (0.1–10 μg/L) showed reduced phagocytic cell recruitment (Brodkin et al. 2007). Neutrophils are the primary phagocytes (Davis et al. 2008) and play an important role in the defense against infections (Tizard 2009). As such, these metamorphs may have reduced immunocompetency.

Independently, exposure to warmer temperatures can produce metamorphs that are immunocompromised (Kohli et al. 2019). That an added stressor such as atrazine, which is ubiquitous in amphibian surface water habitats, can exacerbate the immune effects of temperature is concerning and warrants continued study.

Overall, temperature was the primary driver of phenotypic change, likely representing a trade‐off between energy for growth versus immunity. Elevated temperatures may increase susceptibility to parasites or predators, both of which can have implications for frog populations. These added stressors represent an important avenue for continued study. Further research should also examine the effects of elevated temperature, both independently and in combination with atrazine, on post‐metamorphs and adult frogs, given life history stages in our study were affected differently. The lack of observed effects by atrazine, independently or in combination with temperature, remains surprising, given that previous studies have found multiple effects of atrazine (at similar concentrations) on *L. pipiens* and other amphibian species (Table 3). As such additional research looking at the effects of atrazine in combination with other environmental factors, and in other amphibian species, is warranted.

### 4.7 Conclusions

Exposure to warmer temperatures produced phenotypically challenged frogs (e.g., accelerated development, reduced activity levels). Tadpoles reared at warmer temperatures may be at greater risk of predation and parasitism. Furthermore, metamorphs reared at warmer temperatures displayed reduced immunocompetency, with these effects magnified when combined with atrazine exposure, possibly increasing susceptibility to disease. Given that amphibians encounter multiple interacting environmental stressors, further research using integrated ecohealth approaches is essential to understand and mitigate the impacts of warming and contaminants on amphibian health and conservation.

## Conflicts of Interest

The authors declare no conflicts of interest.

## Supporting information

SI_revised_Clean_version_JEZ__R1.

Master_Excel_Gavel_et_al.

## Data Availability

The data that support the findings of this study are available in the supporting material of this article.
