# Supplementary material for: The Individual and Combined Effects of Warming and Atrazine on Lithobates pipiens Phenotypes: Implications for Frog Declines
Source: J Exp Zool A Ecol Integr Physiol. 2025 Dec 8;345(3):238–58. doi: 10.1002/jez.70054 (PMC12969970; doi:10.1002/jez.70054)
Supplement: Supplementary file 1 — SI_revised_Clean_version_JEZ__R1. [file JEZ-345-238-s002.docx]

**The individual and combined effects of warming and atrazine on *Lithobates pipiens* phenotypes: Implications for frog declines**

Melody J. Gavel^1^, Mark R. Forbes^1^, Derek D.N. Smith^2^, Julia Darabaner^2^, Yol Monica Reyes^2^, Zintis Stasko^2^, David J. Carpenter^2^, Stacey A. Robinson^1,2*^

^1^Department of Biology, Carleton University, Ottawa, Ontario, Canada

^2^Ecotoxicology and Wildlife Health Division, Wildlife and Landscape Science Directorate, Science and Technology Branch, Environment and Climate Change Canada, Ottawa, Ontario, Canada

**Supporting Information**

*Atrazine Herbicide:*

We collected water from a rain collection bucket after every major rainfall, to monitor for any potential input of atrazine from rainfall that may have inadvertently entered mesocosms throughout the experimental period (e.g. when feeding tadpoles on rainy days –Table S12). Water samples were collected using amber glass bottles, and bottles were rinsed with sample water three times before collecting the water sample midway in the water column.

Atrazine samples were kept in the dark at 4 °C until their analysis in May/July 2023. The samples were analyzed at the National Wildlife Research Centre, Environment and Climate Change Canada (Ottawa, ON, Canada), by Laboratory Services. Atrazine samples were prepared for chemical analysis by diluting aliquots with methanol, for final concentrations of < 1 μg/L. A Millex® HV 4 mm syringe filter – 0.45 μm PVDF membrane was then used to filter samples, before they were transferred into a 2 mL auto-sampler vial. The atrazine samples were detected using an AB Sciex Turbo V^TM^ Ion Source and API 5000 Triple Quadrapole Mass Spectrometer with a TurboSpray ion source in positive polarity using multiple reaction monitoring. For the triple quadropole, Curtain Gas was set to 50, Gas 1 and 2 were set to 70, IonSpray Voltage (IS) was set to 2000 V, temperature was set to 700 °C, interface heater was on, collision gas was set to 8, declustering potential was set to 160, and entrance potential was set to 10 V.

Analytical standards were purchased from ChemService (PS-380) and from CDN Isotopes and included 98 % pure Atrazine standard, and a d5-Atrazine (ethyl-D5), respectfully. Calibration curves (R > 0.995) were built using eight concentrations (0.005 to 1 μg/L). The calibration curves were injected before each set of samples, and the internal standard method was used in the calculations. To quantify, the peak Atrazine-a corrected for recoveries of the IS peak “d5-Atrazine-a” was used.

To monitor cross-contamination, two to three solvent blanks (H20:MeOH 50:50) were injected before and after each sample and calibration standard. Furthermore, a sample blank (H20:MeOH 50:50; spiked with IS solution) was analyzed with each sample set. To ensure precision, one random sample per set was analyzed in duplicate. To ensure accuracy, a sample blank (H20:MeOH 50:50; spiked with 2000V IS and standard reference material solution) was analyzed with each set. To ensure calibration accuracy, a second-source atrazine standard solution, purchased from Supelco, was used. Commercially prepared sample aliquots were analyzed with samples to account for variation between sampling days.

The method detection limit was 0.0005 μg/L, and the method reporting limit was 0.002 μg/L. The percent difference between duplicates for results above the method reporting limit was 15 %, demonstrating good method precision. The recovery of standards was between 85-115 %, demonstrating good method accuracy. The recovery for quality control samples ranged from 90 to 94.3 %.

*Metamorph Locomotor Performance:*

The dimensions of our locomotor arena were modified to 1.5’ x 12’ x 2’, as our assays were completed on newly metamorphosed frogs, as compared to adults (Figure S4). The 2’ height was found to be acceptable to ensure metamorphs could not escape the arena. The 12’ length was used to ensure the arena would fit into the temperature-controlled room and to give enough space for metamorphs to perform at least 11 jumps (according to mean jumping distance of newly-metamorphosed *Rana pipiens* in Heinen & Hammond (1997)). The interior of the locomotor arena was lined with waterproof cardboard covered in black plastic. All seams were covered in black masking tape. The farthest end of the arena was covered in clear plastic, and a large patch of grass was placed on the exterior side of the plastic. We used brown paper to line the bottom of the arena according to Zug (1975), and changed the paper between each metamorph.

To perform the assay, the metamorph had its hind legs, feet and posterior abdomen dipped in a 50-50 mixture of dechloraminated water and non-toxic black paint. The metamorph was then released into the locomotor arena, on the side opposite of the grass patch.

Table S1: Variables measured and results at tadpole and metamorph developmental stages in *Lithobates pipiens* exposed to either atrazine (2 μg/L) or dechloraminated water and exposure to either warmed or ambient temperatures. Temperature treatment predictions are referencing warmed treatments, as compared to ambient treatments, and atrazine treatments are referencing atrazine treatments, as compared to control (dechloraminated) treatments. The interaction refers to the interaction between temperature and atrazine. Arrows pointed up indicate a significant (p < 0.05) increase in the variable, whereas arrows pointed down indicate a decrease in the variable. Horizontal lines indicate no effect.

**Development Variable Treatment**

**Stage Temperature Atrazine Interaction**

Tadpole Survival ⎯ ⎯ ⎯

Snout to vent length ↑ ⎯ ⎯

Gosner stage ↑ ⎯ ↓

Locomotor activity ↓ ⎯ ⎯

Startle response ⎯ ⎯ ⎯

Erythrocytes ⎯ ⎯ ⎯

Leukocytes ↑ ⎯ ⎯

Leukocyte/ erythrocyte ⎯ ⎯ ⎯

Neutrophil/lymphocyte ↓ ⎯ ⎯

Eosinophil/leukocyte ⎯ ⎯ ⎯

Monocyte/leukocyte ⎯ ⎯ ⎯

Metamorph Survival ↓ ⎯ ⎯

Days to metamorphosis ↓ ⎯ ⎯

Proportion metamorphosed ⎯ ⎯ ⎯

Proportion female ⎯ ⎯ ⎯

Body mass ↓ ⎯ ⎯

Hepatosomatic index ⎯ ⎯ ⎯

Snout to vent length ↓ ⎯ ⎯

Femur length ↓ ⎯ ⎯

Tibiofibular length ⎯ ⎯ ⎯

Mean distance jumped ⎯ ⎯ ⎯

Maximum distance jumped ⎯ ⎯ ⎯

Mean prods to jump ⎯ ⎯ ⎯

Proportion failed locomotor ⎯ ⎯ ⎯

Glucose ↑ ⎯ ⎯

Bacteria killing ability ⎯ ⎯ ⎯

Erythrocytes ⎯ ⎯ ⎯

Leukocytes ⎯ ⎯ ⎯

Leukocyte/erythrocyte ↓ ⎯ ⎯

Neutrophil/lymphocyte ⎯ ⎯ ↑

Eosinophil/leukocyte ⎯ ⎯ ⎯

Monocyte/leukocyte ↑ ⎯ ⎯

Table S2: Comparisons of general(ized) linear and mixed models and their associated AIC values for the tadpole endpoints. Tank was nested within block as a random effect for each model, except for models wherein these random effects did not add any variation to the model (singular fit). The best fit models are bolded.

Model AIC ΔAIC

**Model 1: Gosner stage**

Model selection not possible with glmmPQL package; presented full model in Table S7 -

**Model 2: Snout to vent length**

1. Temperature*Pesticide + (1|Block/Tank) 1433.48

**Model 3: Erythrocyte counts**

Model selection not possible with glmmPQL package; presented full model in Table S7. -

**Model 4: Leukocyte counts**

Model selection not possible with glmmPQL package; presented full model in Table S7. -

Table S3: Comparisons of general(ized) linear and mixed models and their associated AIC values for the Gosner stage 46 metamorph endpoints. Tank was nested within block as a random effect for each model, except for models wherein these random effects did not add any variation to the model (singular fit). The best fit models are bolded.

Model AIC ΔAIC

**Model 1: Survival**

1. Temperature*Pesticide + (1|Block) 68.61^a^

**Model 2: Days to metamorphosis**

Model selection not possible with glmmPQL package; presented full model in Table S5. -

**Model 3: Sex**

1. Temperature*Pesticide 77.65^b^

**Model 4: Body mass**

1. Temperature*Pesticide + (1|Block/Tank) 410.56

**Model 5: Hepatosomatic index**

1. Temperature*Pesticide + (1|Block/Tank) 290.21 0.59
2. Temperature*Pesticide + Sex + (1|Block/Tank) **289.62**

**Model 6: Snout to vent length**

1. Temperature*Pesticide + (1|Block/Tank) 1550.41

**Model 7: Femur Length**

1. Temperature*Pesticide + Snout to vent length + (1|Block/Tank) 819.35

Table S3: Continued.

Model AIC ΔAIC

**Model 8: Tibiofibular Length**

1. Temperature*Pesticide + Snout to vent length + (1|Block/Tank) 754.44

**Model 9: Mean distance jumped**

1. Temperature*Pesticide + Snout to Vent Length + Femur + Tibiofibular + (1|Block/Tank) -275.88 -30.97
2. Temperature*Pesticide + Femur + Tibiofibular + (1|Block/Tank) -286.13 -20.72
3. Temperature*Pesticide + Snout to Vent Length + Tibiofibular + (1|Block/Tank) -283.32 -23.53
4. Temperature*Pesticide + Snout to Vent Length + (1|Block/Tank) -292.71 -14.14
5. Temperature*Pesticide + Femur + Tibiofibular + (1|Block/Tank) -286.13 -20.72
6. Temperature*Pesticide + Tibiofibular + (1|Block/Tank) -295.76 -11.09
7. Temperature*Pesticide + Femur + (1|Block/Tank) -293.43 -13.42
8. Temperature*Pesticide + (1|Block/Tank)  **-306.85**

**Model 10: Maximum distance jumped**

1. Temperature*Pesticide + Snout to Vent Length + Femur + Tibiofibular + (1|Block/Tank) -183.58 -26.46
2. Temperature*Pesticide + Femur + Tibiofibular + (1|Block/Tank) -190.12 -19.92
3. Temperature*Pesticide + Snout to Vent Length + Tibiofibular + (1|Block/Tank) -192.08 -17.96
4. Temperature*Pesticide + Snout to Vent Length + (1|Block/Tank) -199.84 -10.20
5. Temperature*Pesticide + Femur + Tibiofibular + (1|Block/Tank) -190.12 -19.92
6. Temperature*Pesticide + Femur + (1|Block/Tank) -198.53 -11.51
7. Temperature*Pesticide + Tibiofibular + (1|Block/Tank) -198.67 -11.37
8. Temperature*Pesticide + (1|Block/Tank) -210.04
9. Temperature*Pesticide + (1|Block) **-210.04^a^**

**Model 11: Pass/Fail locomotor trial**

1. Temperature*Pesticide 56.77^b^

Table S3: Continued.

Model AIC ΔAIC

**Model 12: Glucose**

1. Temperature*Pesticide + Body mass + (1|Block/Tank) 410.18

**Model 13: Bacteria Killing Assay**

1. Temperature*Pesticide + (1|Block) + (1|Assay) 403.97 10.49
2. Temperature*Pesticide + (1|Assay) 402.13 8.65
3. Temperature*Pesticide + Body mass + (1|Assay) **393.48^c^**

**Model 14: Erythrocyte counts**

Model selection not possible with glmmPQL package; presented full model in Table S5. -

**Model 15: Leukocyte Counts**

Model selection not possible with glmmPQL package; presented full model in Table S5. -

**Model 16: Leukocyte/Erythrocyte Ratio**

1. Temperature*Pesticide + (1|Block/Tank) -457.60 2.00
2. Temperature*Pesticide + (1|Block) **-459.60^a^**

^a^ Tank removed as it was adding no variation (singular fit).
^b^ All random effects removed as they were adding no variation (singular fit).
^c^ Block removed as it was adding no variation (singular fit).

Table S4: Mean (+/- standard deviation) concentrations of atrazine (µg/L) by treatment and sampling date. Method detection limit (MDL) for atrazine was 0.0005 µg/L.

**Date Ambient Control Warmed Control Ambient Atrazine Warmed Atrazine**

May 17^a^  <MDL <MDL 2.74 (0.26) 2.67 (0.14)

May 19^b^ <MDL <MDL 2.38 2.54

May 26^b^ 0.002 0.001 2.72 2.82

May 30^ac^ 0.002 (0.001) 0.002 (0.002) 2.70 (0.06) 2.65 (0.08)

June 2^b^ <MDL <MDL 2.54 2.66

June 9^b^ <MDL <MDL 2.48 2.56

June 16^b^ 0.0005 <MDL 2.40 2.30

June 23^b^ <MDL <MDL 2.12 1.98

June 30^b^ <MDL <MDL 1.67 1.46

July 7^a^ <MDL <MDL 1.16 (0.04) 1.19 (0.09)

July 14^b^ <MDL <MDL 0.96 1.25

July 17^a^ <MDL <MDL 0.98 (0.07) 1.24 (0.10)

^a^ Sampled from all treatments across all blocks (N = 20 total).

^b^ Sampled from treatments in replicate block 3 only (N = 4 total).

^c^ Date week 2 endpoint collection began.

Table S5: Results of GLM(M) models for *Lithobates* *pipiens* metamorphs exposed to either atrazine (2 μg/L) or dechloraminated water that was either warmed or ambient. Random effects are denoted by 1|Block/Tank or 1|Assay. Sample size is denoted by (n). Significant effects (p < 0.05) have been bolded.

Model β Estimate ± SE *t* or *z* p value Variance SD n

Model 1: **Survival** ~ Temperature*Pesticide +

(1|Block) 717

Fixed Effects (Intercept) 3.472 ± 0.475 7.312  **2.64 x 10^-13^**

Treatment (Reference: Ambient)

Warmed -1.086 ± 0.523 -2.076  **0.038**

Treatment (Reference: Dechloraminated)

Atrazine 0.200 ± 0.686 0.292 0.770

Interaction 0.488 ± 0.807 0.605 0.545

Random Effects

Block 0.053 0.230

Model 2: **Days** **to metamorphosis** ~ Temperature*Pesticide +

(1|Block/Tank) 698

Fixed Effects (Intercept) 4.059 ± 0.007 546.692  **0.000**

Treatment (Reference: Ambient)

Warmed -0.154 ± 0.010 -15.350  **0.000**

Treatment (Reference: Dechloraminated)

Atrazine -0.014 ± 0.010 -1.374 0.197

Interaction 0.014 ± 0.014 0.988 0.344

Random Effects

Block 1.453 x 10^-6^

Block/Tank 0.013

Table S5: Continued.

Model β Estimate ± SE *t* or *z* p value Variance SD n

Model 3: **Sex** ~ Temperature*Pesticide 179

Fixed Effects (Intercept) -0.258 ± 0.323 -0.798 0.425

Treatment (Reference: Ambient)

Warmed 0.097 ± 0.430 0.227 0.821

Treatment (Reference: Dechloraminated)

Atrazine 0.158 ± 0.452 0.349 0.727

Interaction -0.403 ± 0.607 -0.664 0.507

Model 4: **Body** **Mass** ~ Temperature*Pesticide + 360

(1|Block/Tank)

Fixed Effects (Intercept) 3.162 ± 0.094 33.621  **6.05 x 10^15^**

Treatment (Reference: Ambient)

Warmed -0.552 ± 0.125 -4.406  **0.001**

Treatment (Reference: Dechloraminated)

Atrazine -0.067 ± 0.133 -0.500 0.624

Interaction -0.006 ± 0.178 0.033 0.974

Random Effects

Block 0.000 0.000 Block/Tank 0.027 0.166

Model 5: **Hepatosomatic index** ~ Temperature*Pesticide + 179

(1|Block/Tank)

Fixed Effects (Intercept) 2.930 ± 0.135 21.634  **1.3 x 10^-13^**

Treatment (Reference: Ambient)

Warmed 0.119 ± 0.167 0.714 0.491

Treatment (Reference: Dechloraminated)

Atrazine 0.056 ± 0.177 0.317 0.757

Sex (Reference: Female) -0.202 ± 0.075 -2.711  **0.007**

Interaction -0.169 ± 0.237 -0.714 0.491

Random Effects

Block 0.006 0.074 Block/Tank 0.039 0.197

Table S5: Continued.

Model β Estimate ± SE *t* or *z* p value Variance SD n

Model 6: **Snout to Vent Length** ~ Temperature*Pesticide +

(1|Block/Tank) 360

Fixed Effects (Intercept) 36.851 ± 0.461 80.022 **0.00 x 10^-16^**

Treatment (Reference: Ambient)

Warmed -2.748 ± 0.614 -4.478  **4.63 x 10^-4^**

Treatment (Reference: Dechloraminated)

Atrazine -0.330 ± 0.651 -0.507 0.620

Interaction 0.014 ± 0.870 0.016 0.988

Random Effects

Block 0.000 0.000 Block/Tank 0.652 0.807

Model 7: **Femur** ~ Temperature*Pesticide + Sex +

+ SVL + (1|Block/Tank) 360

Fixed Effects (Intercept) 6.111 ± 0.703 8.697  **0.00 x 10^-16^**

Treatment (Reference: Ambient)

Warmed -1.233 ± 0.180 -6.854  **2.88 x 10^-6^**

Treatment (Reference: Dechloraminated)

Atrazine -0.247 ± 0.182 -1.356 0.197

SVL 0.212 ± 0.019 11.326 **0.00 x 10^-16^**

Interaction 0.276 ± 0.244 1.132 0.276

Random Effects

Block 5.343 x 10^-11^ 7.309 x 10^-6^ Block/Tank 0.041 0.204

Table S5: Continued.

Model β Estimate ± SE *t* or *z* p value Variance SD n

Model 8: **Tibiofibular** ~ Temperature*Pesticide + SVL + 360

+ (1|Block/Tank)

Fixed Effects (Intercept) 0.383 ± 0.631 0.607 0.544

Treatment (Reference: Ambient)

Warmed -0.086 ± 0.133 -0.643 0.528

Treatment (Reference: Dechloraminated)

Atrazine -0.099 ± 0.132 -0.754 0.463

SVL 0.385 ± 0.017 22.739  **0.00 x 10^-`6^**

Interaction 0.071 ± 0.177 0.403 0.693

Random Effects

Block 0.000 0.000 Block/Tank 0.013 0.116

Model 9: **Mean distance jumped** ~ Temperature*Pesticide + 139 (1|Block/Tank)

Fixed Effects (Intercept) 0.263 ± 0.016 16.736  **1.85 x 10^-10^**

Treatment (Reference: Ambient)

Warmed 0.010 ± 0.021 0.483 0.636

Treatment (Reference: Dechloraminated)

Atrazine 0.008 ± 0.023 0.353 0.729

Interaction -0.009 ± 0.031 -0.309 0.761

Random Effects

Block 0.000 0.000

Block/Tank 4.391 x 10^-4^ 0.068

Table S5: Continued.

Model β Estimate ± SE *t* or *z* p value Variance SD n

Model 10: **Maximum distance jumped** ~ 175

Temperature*Pesticide + (1|Block/Tank)

Fixed Effects (Intercept) 0.392 ± 0.020 19.412 **0.000 x 10^-16^**

Treatment (Reference: Ambient)

Warmed -0.006 ± 0.03 0.250 0.803

Treatment (Reference: Dechloraminated)

Atrazine -0.010 ± 0.027 -0.356 0.722

Interaction -0.001 ± 0.037 -0.026 0.979

Random Effects

Block 0.0002 0.01

Model 11: **Pass/Fail Locomotor** ~ Temperature*Pesticide 175

Fixed Effects (Intercept) 1.946 ± 0.478 4.070  **4.7 x 10^-5^**

Treatment (Reference: Ambient)

Warmed -0.203 ± 0.630 -0.322 0.747

Treatment (Reference: Dechloraminated)

Atrazine -0.709 ± 0.610 -1.163 0.245 Interaction 0.037 ± 0.807 0.045 0.964

Model 12: **Glucose** ~ Temperature*Pesticide + Body mass 166

+ (1|Block/Tank)

Fixed Effects (Intercept) 0.033 ± 0.499 0.065 0.948

Treatment (Reference: Ambient)

Warmed 0.190 ± 0.263 3.314  **0.004**

Treatment (Reference: Dechloraminated)

Atrazine 0.190 ± 0.260 0.730 0.477

Body mass 0.535 ± 0.146 3.659  **3.43 x 10^-4^** Interaction -0.052 ± 0.348 14.054 0.884

Random Effects

Block 0.000 0.000

Block/Tank 0.073 0.271

Table S5: Continued.

Model β Estimate ± SE *t* or *z* p value Variance SD n

Model 13: **Bacteria killing ability** ~ Temperature*Pesticide 49

+ (1 | Assay)

Fixed Effects (Intercept) 41.813 ± 6.731 6.212  **0.001**

Treatment (Reference: Ambient)

Warmed -7.973 ± 5.889 -1.354 0.183

Treatment (Reference: Dechloraminated)

Atrazine 1.035 ± 6.050 -0.171 0.865

Interaction 0.349 ± 9.115 42.030 0.970

Random Effects

Assay 109.14 10.447

Block 9.69 3.113

Model 14: **Erythrocytes**~ Temperature*Pesticide + 88

+ (1|Block/Tank)

Fixed Effects (Intercept) 8.407 ± 0.078 107.147  **0.000**

Treatment (Reference: Ambient)

Warmed -0.009 ± 0.104 -0.082 0.936

Treatment (Reference: Dechloraminated)

Atrazine -0.035 ± 0.110 0.321 0.755 Interaction -0.074 ± 0.147 -0.499 0.629

Random Effects

Block 1.632 x 10^-6^

Block/Tank 1.682 x 10^-5^

Table S5: Continued.

Model β Estimate ± SE *t* or *z* p value Variance SD n

Model 15: **Leukocytes** ~ Temperature*Pesticide + 88

+ (1|Block/Tank)

Fixed Effects (Intercept) 5.001 ± 0.115 43.552  **0.000**

Treatment (Reference: Ambient)

Warmed -0.305 ± 0.148 -2.060 0.066

Treatment (Reference: Dechloraminated)

Atrazine -0.130 ± 0.153 -0.852 0.414 Interaction 0.192 ± 0.212 0.904 0.387

Random Effects

Block 0.105

Block/Tank 2.530 x 10^-5^

Model 16: **Leukocytes/Erythrocytes** ~ Temperature*Pesticide + 88

+ (1|Block)

Fixed Effects (Intercept) 0.036 ± 0.003 10.284  **2.9 x 10^-10^**

Treatment (Reference: Ambient)

Warmed -0.010 ± 0.004 -2.328  **0.022**

Treatment (Reference: Dechloraminated)

Atrazine -0.005 ± 0.004 -1.221 0.226 Interaction 0.007 ± 0.006 1.212 0.239

Random Effects

Block 1.127 x 10^-5^ 0.003

Table S5: Continued.

Model β Estimate ± SE *t* or *z* p value Variance SD n

Model 17: **Temperature^a^** ~ Temperature Treatment + 18008
(1|Date) + (1|Block)
Fixed Effects (Intercept) 20.05 ± 0.478 42.32  **0.00 x 10^-16^**

Treatment (Reference: Ambient)

Warmed 2.402 ± 0.023 103.04  **0.00 x 10^-16^**

Random Effects

Date 13.757 3.709

Block 0.011 0.105

^a^ Temperature was compared between warmed and ambient treatments over the experimental period, using temperature readings from HOBO dataloggers.

Table S6: Results of the Kruskal-Wallis and Posthoc Dunn’s test (Benjamini-Hochberg correction applied) for *Lithobates* *pipiens* tadpoles sampled after two weeks of exposure to either atrazine (2 μg/L) or dechloraminated water that was either warmed (Warmed Control, Warmed Atrazine) or ambient (Ambient Control, Ambient Atrazine). Locomotor performance results are reported before (pre) and after (post) the startle. Significant effects (p < 0.05) are bolded.

**Endpoint Treatments X^2^ p-value Z Unadjusted p Adjusted p**

**Survival**  5.506 0.138

Ambient Control / Warmed Control 0.000 1.000 1.000

Ambient Control / Ambient Atrazine 0.000 1.000 1.000

Warmed Control / Ambient Atrazine 0.000 1.000 1.000

Ambient Control / Warmed Atrazine 1.841 0.066 0.131

Warmed Control / Warmed Atrazine 1.952 0.051 0.305

Ambient Atrazine / Warmed Atrazine 1.841 0.066 0.197

**Proportion Startled**  1.126 0.771

Ambient Control / Warmed Control 0.437 0.662 0.993

Ambient Control / Ambient Atrazine 0.691 0.489 1.000

Warmed Control / Ambient Atrazine 0.292 0.771 0.771

Ambient Control / Warmed Atrazine 1.020 0.308 1.000

Warmed Control / Warmed Atrazine 0.618 0.536 1.000

Ambient Atrazine / Warmed Atrazine 0.292 0.771 0.924

Table S6: Continued.

**Endpoint Treatments X^2^ p-value Z Unadjusted p Adjusted p**

**Locomotor Activity** 40.008 **1.254 x 10^-6^**

Ambient Control -post / Ambient Control -pre -0.676 0.499 0.607

Ambient Control -post / Warmed Control -post 1.670 0.095 0.166

Ambient Control -pre / Warmed Control -post 2.383  **0.017 0.040**

Ambient Control -post / Warmed Control -pre 2.811  **0.005 0.014**

Ambient Control -pre / Warmed Control -pre 3.523  **4.261 x 10^-4^ 0.002**

Warmed Control -post / Warmed Control -pre 1.210 0.226 0.317

Ambient Control -post / Ambient Atrazine -post -1.281 0.200 0.295

Ambient Control -pre / Ambient Atrazine -post -0.605 0.545 0.636

Warmed Control -post / Ambient Atrazine -post -3.021  **0.003 0.009**

Warmed Control -pre / Ambient Atrazine -post -4.161 **3.161 x 10^-5^**  **4.426 x 10^-4^**

Ambient Control -post / Ambient Atrazine -pre -1.773 0.076 0.152

Ambient Control -pre / Ambient Atrazine -pre -1.097 0.273 0.347

Warmed Control -post / Ambient Atrazine -pre -3.539  **4.023 x 10^-4^ 0.003**

Warmed Control -pre / Ambient Atrazine -pre -4.679 **2.878 x 10^-6^** **8.057 x 10^-5^**

Ambient Atrazine -post / Ambient Atrazine -pre -0.491 0.623 0.698

Ambient Control -post / Warmed Atrazine -post 1.543 0.123 0.202

Ambient Control -pre / Warmed Atrazine -post 2.256 **0.024** 0.052

Warmed Control -post / Warmed Atrazine -post -0.134 0.894 0.927

Warmed Control -pre / Warmed Atrazine -post -1.343 0.179 0.279

Ambient Atrazine -post / Warmed Atrazine -post 2.895  **0.004 0.012**

Ambient Atrazine -pre / Warmed Atrazine -post 3.412  **0.001 0.003**

Ambient Control -post / Warmed Atrazine -pre 1.733 0.083 0.155

Ambient Control -pre / Warmed Atrazine -pre 2.446  **0.014 0.037**

Warmed Control -post / Warmed Atrazine -pre 0.067 0.946 0.946

Warmed Control -pre / Warmed Atrazine -pre -1.143 0.253 0.337

Ambient Atrazine -post / Warmed Atrazine -pre 3.084  **0.002 0.008**

Ambient Atrazine -pre / Warmed Atrazine -pre 3.602  **3.159 x 10^-4^ 0.003**

Warmed Atrazine -post / Warmed Atrazine -pre 0.201 0.841 0.905

Table S6: Continued.

**Endpoint Treatments X^2^ p-value Z Unadjusted p Adjusted p**

**Leukocyte/Erythrocyte Ratio** 8.317 **0.004**

Ambient Control / Warmed Control -2.281  **0.023** 0.135

Ambient Control / Ambient Atrazine -0.454 0.650 0.780

Warmed Control / Ambient Atrazine 1.779 0.075 0.150

Ambient Control / Warmed Atrazine -2.241 **0.025** 0.075

Warmed Control / Warmed Atrazine 0.090 0.928 0.928

Ambient Atrazine / Warmed Atrazine -1.729 0.084 0.126

**Neutrophil/Lymphocyte Ratio** 20.051  **1.656 x 10^-4^**

Ambient Control / Warmed Control 2.796  **0.005 0.010**

Ambient Control / Ambient Atrazine -1.544 0.123 0.147

Warmed Control / Ambient Atrazine -4.288  **1.805 x 10^-5^ 1.08 x 10^-4^**

Ambient Control / Warmed Atrazine 1.274 0.203 0.203

Warmed Control / Warmed Atrazine -1.633 0.102 0.154

Ambient Atrazine / Warmed Atrazine 2.848  **0.004 0.013**

**Eosinophil/Leukocyte Ratio** 2.308 0.369

Ambient Control / Warmed Control -1.515 0.130 0.779

Ambient Control / Ambient Atrazine -0.842 0.400 1.000

Warmed Control / Ambient Atrazine 0.602 0.547 0.657

Ambient Control / Warmed Atrazine -0.800 0.424 0.847

Warmed Control / Warmed Atrazine 0.771 0.441 0.661

Ambient Atrazine / Warmed Atrazine 0.108 0.914 0.914

**Monocyte/Leukocyte Ratio** 3.149 0.369

Ambient Control / Warmed Control -0.425 0.671 0.671

Ambient Control / Ambient Atrazine -1.625 0.104 0.625

Warmed Control / Ambient Atrazine -1.165 0.244 0.488

Ambient Control / Warmed Atrazine -1.194 0.232 0.697

Warmed Control / Warmed Atrazine -0.709 0.478 0.717

Ambient Atrazine / Warmed Atrazine 0.548 0.584 0.701

Table S7: Results of GLM(M) models for *Lithobates* *pipiens* tadpoles sampled after two weeks of exposure to either atrazine (2 μg/L) or dechloraminated water that was either warmed or ambient. Random effects are denoted by 1|Block/Tank. Sample size is denoted by (n). Significant effects (p < 0.05) have been bolded.

Model β Estimate ± SE *t* or *z* p value Variance SD n

Model 1: **Gosner stage** ~ Temperature*Pesticide + 377

(1|Block/Tank)

Fixed Effects (Intercept) 3.273 ± 0.010 315.238 **0.000**

Treatment (Reference: Ambient)

Warmed 0.045 ± 0.009 5.093  **0.001**

Treatment (Reference: Dechloraminated)

Atrazine 0.010 ± 0.009 1.041 0.322

Interaction -0.034 ± 0.012 -2.753  **0.020**

Random Effects

Block 0.018

Block/Tank 0.012

Model 2: **Snout to vent length** ~ Temperature*Pesticide + 377

(1|Block/Tank)

Fixed Effects (Intercept) 11.662 ± 0.724 16.119  **2.51 x 10^-6^**

Treatment (Reference: Ambient)

Warmed 2.399 ± 0.492 4.873  **0.001**

Treatment (Reference: Dechloraminated)

Atrazine 0.324 ± 0.531 0.610 0.555

Interaction -1.172 ± 0.700 -1.675 0.124

Random Effects

Block 1.925 1.388

Block/Tank 0.410 0.640

Table S7: Continued.

Model β Estimate ± SE *t* or *z* p value Variance SD n

Model 3: **Erythrocytes** ~ Temperature*Pesticide + 125

Snout to vent length + (1|Block/Tank)

Fixed Effects (Intercept) 7.017 ± 0.302 23.232  **0.001**

Treatment (Reference: Ambient)

Warmed 0.331 ± 0.148 2.234 0.050

Treatment (Reference: Dechloraminated)

Atrazine 0.099 ± 0.153 0.646 0.533

Snout to vent Length 0.028 ± 0.023 1.202 0.232

Interaction -0.255 ± 0.198 -1.285 0.228

Random Effects

Block 0.047

Block/Tank 3.422 x 10^-5^

Model 4: **Leukocytes** ~ Temperature*Pesticide + 125

Gosner stage + (1|Block/Tank)

Fixed Effects (Intercept) 0.619 ± 1.843 0.336 0.748

Treatment (Reference: Ambient)

Warmed 0.562 ± 0.213 2.639  **0.025**

Treatment (Reference: Dechloraminated)

Atrazine 0.096 ± 0.226 0.424 0.680

Gosner stage 0.120 ± 0.069 1.744 0.084

Interaction -0.147 ± 0.282 -0.523 0.613

Random Effects

Block 8.309 x 10^-6^ Block/Tank 1.595 x 10^-5^

Table S8: Results of pairwise comparisons of GLM(M) models for *Lithobates* *pipiens* tadpoles sampled after two weeks of exposure to either atrazine (2 μg/L) or dechloraminated water that was either warmed or ambient. Significant effects (p < 0.05) have been bolded. Sample size is denoted by (n).

Model Pairwise Comparison Odds Ratio / Estimate ± SE t or z p value n

Model 1: **Gosner stage** ~ Temperature*Pesticide + 377

(1|Block/Tank)

Ambient Control / Warmed Control 0.965 ± 0.008 -5.093  **0.002**

Ambient Control / Ambient Atrazine 0.999 ± 0.009 -1.041 0.730

Ambient Control / Warmed Atrazine 0.989 ± 0.009 -2.301 0.162

Warmed Control / Ambient Atrazine 1.044 ± 0.009 3.974  **0.012**

Warmed Control / Warmed Atrazine 1.034 ± 0.008 3.012 0.054

Ambient Atrazine / Warmed Atrazine 0.998 ± 0.009 -1.180 0.652

Model 2: **Snout to vent length** ~ Temperature*Pesticide + 377

(1|Block/Tank)

Ambient Control / Warmed Control -2.399 ± 0.493 -4.862  **0.003**

Ambient Control / Ambient Atrazine -0.324 ± 0.534 -0.607 0.928

Ambient Control / Warmed Atrazine -1.550 ± 0.493 -3.143  **0.043**

Warmed Control / Ambient Atrazine 2.075 ± 0.493 4.209  **0.008**

Warmed Control / Warmed Atrazine 0.848 ± 0.456 1.860 0.303

Ambient Atrazine / Warmed Atrazine -1.227 ± 0.493 -2.488 0.122

Model 4: **Erythrocytes** ~ Temperature*Pesticide + 125

Snout to vent length + (1|Block/Tank)

Ambient Control / Warmed Control 189 ± 28.0 -2.234 0.179

Ambient Control / Ambient Atrazine 239 ± 36.4 -0.646 0.915

Ambient Control / Warmed Atrazine 221 ± 31.9 -1.211 0.634

Warmed Control / Ambient Atrazine 332 ± 48.8 1.582 0.430

Warmed Control / Warmed Atrazine 308 ± 38.9 1.236 0.620

Ambient Atrazine / Warmed Atrazine 244 ± 35.0 -0.531 0.950

Table S8: Continued.

Model Pairwise Comparison Odds Ratio / Estimate ± SE t or z p value n

Model 5: **Leukocytes** ~ Temperature*Pesticide +

Gosner stage + (1|Block/Tank) 125

Ambient Control / Warmed Control 10.3 ± 2.19 -2.639 0.097

Ambient Control / Ambient Atrazine 16.4 ± 3.72 -0.424 0.973

Ambient Control / Warmed Atrazine 10.8 ± 2.16 -2.565 0.109

Warmed Control / Ambient Atrazine 28.8 ± 6.06 2.215 0.184

Warmed Control / Warmed Atrazine 19.0 ± 3.18 0.307 0.989

Ambient Atrazine / Warmed Atrazine 11.9 ± 2.35 -2.106 0.216

Table S9: Results of pairwise comparisons of GLM(M) models for *Lithobates* *pipiens* metamorphs exposed to either atrazine (2 μg/L) or dechloraminated water that was either warmed (Warmed Control, Warmed Atrazine) or ambient (Ambient Control, Ambient Atrazine). Significant effects (p < 0.05) have been bolded. Sample size is denoted by (n).

Model Pairwise Comparison Odds Ratio / Estimate ± SE t or z p value n

Model 1: **Survival** ~ Temperature*Pesticide + 717

(1|Block)

Ambient Control / Warmed Control 3.040 ± 1.589 2.076 0.161

Ambient Control / Ambient Atrazine 0.840 ± 0.576 -0.292 0.991

Ambient Control / Warmed Atrazine 1.527 ± 0.870 0.698 0.898

Warmed Control / Ambient Atrazine 0.284 ± 0.161 -2.264 0.107

Warmed Control / Warmed Atrazine 0.516 ± 0.219 -1.619 0.368

Ambient Atrazine / Warmed Atrazine 1.866 ± 1.142 0.976 0.763

Model 2: **Days** **to metamorphosis** ~ Temperature*Pesticide + 698

(1|Block/Tank)

Ambient Control / Warmed Control 1.236 ± 0.012 15.35  **0.000**

Ambient Control / Ambient Atrazine 1.075 ± 0.011 1.374 0.539

Ambient Control / Warmed Atrazine 1.236 ± 0.012 15.345  **0.000**

Warmed Control / Ambient Atrazine 0.921 ± 0.009 -13.916  **0.000**

Warmed Control / Warmed Atrazine 1.060 ± 0.010 0.040 1.000

Ambient Atrazine / Warmed Atrazine 1.218 ± 0.012 13.916  **0.000**

Table S9: Continued.

Model Pairwise Comparison Odds Ratio / Estimate ± SE t or z p value n

Model 3: **Sex** ~ Temperature*Pesticide 179

Ambient Control / Warmed Control 0.907 ± 0.390 -0.227 0.996

Ambient Control / Ambient Atrazine 0.854 ± 0.386 -0.349 0.985

Ambient Control / Warmed Atrazine 1.159 ± 0.502 0.341 0.986

Warmed Control / Ambient Atrazine 0.942 ± 0.400 -0.142 0.999

Warmed Control / Warmed Atrazine 1.278 ± 0.517 0.606 0.930

Ambient Atrazine / Warmed Atrazine 1.357 ± 0.581 0.713 0.892

Model 4: **Body** **Mass** ~ Temperature*Pesticide + 360

(1|Block/Tank)

Ambient Control / Warmed Control 0.552 ± 0.128 4.327  **0.006**

Ambient Control / Ambient Atrazine 0.067 ± 0.138 0.483 0.961

Ambient Control / Warmed Atrazine 0.613 ± 0.128 4.772  **0.003**

Warmed Control / Ambient Atrazine -0.486 ± 0.127 -3.808  **0.013**

Warmed Control / Warmed Atrazine 0.061 ± 0.118 0.512 0.955

Ambient Atrazine / Warmed Atrazine 0.546 ± 0.128 4.257  **0.007**

Model 5: **Hepatosomatic index**  ~ Temperature*Pesticide + 179

(1|Block/Tank)

Ambient Control / Warmed Control -0.119 ± 0.170 -0.703 0.894

Ambient Control / Ambient Atrazine -0.057 ± 0.183 -0.306 0.990

Ambient Control / Warmed Atrazine -0.006 ± 0.170 -0.037 1.000

Warmed Control / Ambient Atrazine 0.063 ± 0.170 0.371 0.982

Warmed Control / Warmed Atrazine 0.113 ± 0.157 0.720 0.887

Ambient Atrazine / Warmed Atrazine 0.050 ± 0.170 0.293 0.991

Table S9: Continued.

Model Pairwise Comparison Odds Ratio / Estimate ± SE t or z p value n

Model 6: **Snout to Vent Length**  ~ Temperature*Pesticide + 360

(1|Block/Tank)

Ambient Control / Warmed Control 2.748 ± 0.625 4.398  **0.005**

Ambient Control / Ambient Atrazine 0.330 ± 0.674 0.489 0.960

Ambient Control / Warmed Atrazine 3.064 ± 0.629 4.874  **0.003**

Warmed Control / Ambient Atrazine -2.418 ± 0.624 -3.873  **0.012**

Warmed Control / Warmed Atrazine 0.316 ± 0.580 0.525 0.946

Ambient Atrazine / Warmed Atrazine 2.734 ± 0.628 4.352  **0.006**

Model 7: **Femur** ~ Temperature*Pesticide + Sex + 360

+ SVL + (1|Block/Tank)

Ambient Control / Warmed Control 1.233 ± 0.183 6.726  **1.0 x 10^-4^**

Ambient Control / Ambient Atrazine 0.247 ± 0.189 1.308 0.577

Ambient Control / Warmed Atrazine 1.204 ± 0.186 6.488 **1.0 x 10^-4^**

Warmed Control / Ambient Atrazine -0.986 ± 0.181 -5.437  **0.001**

Warmed Control / Warmed Atrazine -0.029 ± 0.163 -0.179 1.000

Ambient Atrazine / Warmed Atrazine 0.957 ± 0.183 5.218  **0.001**

Model 8: **Tibiofibular** ~ Temperature*Pesticide + SVL + 360

+ (1|Block/Tank)

Ambient Control / Warmed Control 0.086 ± 0.136 0.630 0.921

Ambient Control / Ambient Atrazine 0.099 ± 0.137 0.727 0.884

Ambient Control / Warmed Atrazine 0.114 ± 0.138 0.825 0.842

Warmed Control / Ambient Atrazine 0.014 ± 0.134 0.102 1.000

Warmed Control / Warmed Atrazine 0.028 ± 0.118 0.239 0.995

Ambient Atrazine / Warmed Atrazine 0.015 ± 0.136 0.107 1.000

Table S9: Continued.

Model Pairwise Comparison Odds Ratio / Estimate ± SE t or z p value n

Model 9: **Mean distance jumped** ~ Temperature*Pesticide + 139

(1|Block/Tank)

Ambient Control / Warmed Control -0.010 ± 0.022 -0.474 0.963

Ambient Control / Ambient Atrazine -0.008 ± 0.023 -0.340 0.986

Ambient Control / Warmed Atrazine -0.009 ± 0.022 -0.395 0.978

Warmed Control / Ambient Atrazine 0.002 ± 0.022 0.101 1.000

Warmed Control / Warmed Atrazine 0.001 ± 0.021 0.071 1.000

Ambient Atrazine / Warmed Atrazine -0.001 ± 0.023 -0.034 1.000

Model 10: **Max distance jumped** ~ Temperature*Pesticide 175

+ (1|Block/Tank)

Ambient Control / Warmed Control 0.006 ± 0.026 0.247 0.995

Ambient Control / Ambient Atrazine 0.010 ± 0.028 0.348 0.986

Ambient Control / Warmed Atrazine 0.017 ± 0.026 0.650 0.916

Warmed Control / Ambient Atrazine 0.003 ± 0.026 0.124 0.999

Warmed Control / Warmed Atrazine 0.011 ± 0.025 0.432 0.973

Ambient Atrazine / Warmed Atrazine 0.007 ± 0.026 0.282 0.992

Model 11: **Pass/Fail Locomotor** ~ Temperature*Pesticide 175

Ambient Control / Warmed Control 1.23 ± 0.771 0.322 0.988

Ambient Control / Ambient Atrazine 2.03 ± 1.239 1.163 0.650

Ambient Control / Warmed Atrazine 2.40 ± 1.400 1.500 0.437

Warmed Control / Ambient Atrazine 1.66 ± 0.926 0.907 0.801

Warmed Control / Warmed Atrazine 1.96 ± 1.036 1.272 0.581

Ambient Atrazine / Warmed Atrazine 1.18 ± 0.597 0.329 0.988

Table S9: Continued.

Model Pairwise Comparison Odds Ratio / Estimate ± SE t or z p value n

Model 12: **Glucose** ~ Temperature*Pesticide + Body mass 166

+ (1|Block/Tank)

Ambient Control / Warmed Control -0.872 ± 0.268 -3.252  **0.027**

Ambient Control / Ambient Atrazine -0.190 ± 0.271 -0.702 0.894

Ambient Control / Warmed Atrazine -1.010 ± 0.266 -3.793  **0.010**

Warmed Control / Ambient Atrazine 0.682 ± 0.268 2.548 0.097

Warmed Control / Warmed Atrazine -0.138 ± 0.232 -0.597 0.931

Ambient Atrazine / Warmed Atrazine -0.820 ± 0.266 -3.083  **0.038**

Model 13: **Bacteria killing ability** ~ Temperature*Pesticide + 49

(1 | Assay)

Ambient Control / Warmed Control 7.97 ± 5.97 1.366 0.545

Ambient Control / Ambient Atrazine -1.03 ± 6.20 -0.167 0.998

Ambient Control / Warmed Atrazine 6.59 ± 6.74 0.977 0.763

Warmed Control / Ambient Atrazine -9.01 ± 6.37 -1.414 0.498

Warmed Control / Warmed Atrazine -1.38 ± 7.04 -0.197 0.997

Ambient Atrazine / Warmed Atrazine 7.62 ± 7.32 1.042 0.726

Model 14: **Erythrocytes** ~ Temperature*Pesticide + 88

+ (1|Block/Tank)

Ambient Control / Warmed Control 253 ± 26.4 0.082 1.000

Ambient Control / Ambient Atrazine 242 ± 26.6 -0.321 0.988

Ambient Control / Warmed Atrazine 263 ± 27.7 0.445 0.973

Warmed Control / Ambient Atrazine 240 ± 24.8 -0.425 0.979

Warmed Control / Warmed Atrazine 261 ± 25.6 0.390 0.979

Ambient Atrazine / Warmed Atrazine 272 ± 28.4 0.788 0.858

Table S9: Continued.

Model Pairwise Comparison Odds Ratio / Estimate ± SE t or z p value n

Model 15: **Leukocytes** ~ Temperature*Pesticide + 88

+ (1|Block/Tank)

Ambient Control / Warmed Control 21.1 ± 3.13 2.060 0.230

Ambient Control / Ambient Atrazine 17.7 ± 2.71 0.852 0.829

Ambient Control / Warmed Atrazine 19.9 ± 2.90 1.671 0.386

Warmed Control / Ambient Atrazine 13.1 ± 2.01 -1.141 0.674

Warmed Control / Warmed Atrazine 14.7 ± 2.15 -0.418 0.974

Ambient Atrazine / Warmed Atrazine 17.5 ± 2.64 0.751 0.874

Model 16: **Leukocytes/Erythrocytes** ~ Temperature*Pesticide + 88

+ (1|Block)

Ambient Control / Warmed Control 0.010 ± 0.004 2.307 0.105

Ambient Control / Ambient Atrazine 0.005 ± 0.005 1.200 0.639

Ambient Control / Warmed Atrazine 0.008 ± 0.004 1.908 0.233

Warmed Control / Ambient Atrazine -0.004 ± 0.004 -1.005 0.747

Warmed Control / Warmed Atrazine -0.002 ± 0.004 -0.437 0.972

Ambient Atrazine / Warmed Atrazine 0.003 ± 0.004 0.606 0.930

Table S10: Results of the Kruskal-Wallis and Posthoc Dunn’s test (Benjamini-Hochberg correction applied) for *Lithobates* *pipiens* metamorphs reared in either atrazine (2 μg/L) or dechloraminated water that was either warmed (Warmed Control, Warmed Atrazine) or ambient (Ambient Control, Ambient Atrazine). Significant effects (p < 0.05) are bolded.

**Endpoint Treatments X^2^ p-value Z Unadjusted p Adjusted p**

**Completion of metamorphosis** 6.487 0.090

Ambient Control / Warmed Control -1.716 0.086 0.172

Ambient Control / Ambient Atrazine 0.203 0.839 1.000

Warmed Control / Ambient Atrazine 1.930 0.054 0.322

Ambient Control / Warmed Atrazine -1.659 0.097 0.146

Warmed Control / Warmed Atrazine 0.060 0.952 0.952

Ambient Atrazine / Warmed Atrazine -1.873 0.061 0.183

**Mean prods to jump** 1.276 0.735

Ambient Control / Warmed Control 0.171 0.864 1.000

Ambient Control / Ambient Atrazine 0.082 0.935 0.935

Warmed Control / Ambient Atrazine -0.086 0.932 1.000

Ambient Control / Warmed Atrazine -0.795 0.427 0.853

Warmed Control / Warmed Atrazine -1.011 0.312 1.000

Ambient Atrazine / Warmed Atrazine -0.880 0.372 1.000

**Jumped to end of runway** 0.126 0.989

Ambient Control / Warmed Control -0.016 0.988 1.000

Ambient Control / Ambient Atrazine -0.295 0.768 1.000

Warmed Control / Ambient Atrazine -0.295 0.768 1.000

Ambient Control / Warmed Atrazine -0.016 0.988 1.000

Warmed Control / Warmed Atrazine 0.000 1.000 1.000

Ambient Atrazine / Warmed Atrazine 0.295 0.768 1.000

Table S10: Continued.

**Endpoint Treatments X^2^ p-value Z Unadjusted p Adjusted p**

**Neutrophil / Lymphocyte** 12.248  **0.007**

Ambient Control / Warmed Control 0.460 0.646 0.657

Ambient Control / Ambient Atrazine 0.864 0.388 0.582

Warmed Control / Ambient Atrazine 0.461 0.644 0.774

Ambient Control / Warmed Atrazine 3.094  **0.002 0.012**

Warmed Control / Warmed Atrazine 2.835  **0.005 0.014**

Ambient Atrazine / Warmed Atrazine 2.173 **0.030** 0.060

**Eosinophil / Leukocyte** 0.483 0.923

Ambient Control / Warmed Control -0.508 0.611 1.000

Ambient Control / Ambient Atrazine 0.109 0.914 1.000

Warmed Control / Ambient Atrazine 0.624 0.533 1.000

Ambient Control / Warmed Atrazine -0.047 0.962 0.962

Warmed Control / Warmed Atrazine 0.500 0.620 1.000

Ambient Atrazine / Warmed Atrazine -0.163 0.870 1.000

**Monocyte / Leukocyte** 8.71 **0.033**

Ambient Control / Warmed Control -2.682  **0.007 0.044**

Ambient Control / Ambient Atrazine -2.287  **0.022** 0.067

Warmed Control / Ambient Atrazine 0.244 0.808 0.808

Ambient Control / Warmed Atrazine -1.255 0.210 0.314

Warmed Control / Warmed Atrazine 1.535 0.125 0.249

Ambient Atrazine / Warmed Atrazine 1.183 0.237 0.284

Table S11: Physicochemical water quality parameters by treatment and sampling date. Water quality was measured in each individual mesocosm on experimental day 0 (May 16^th^, 2023). Values from these sampling dates are reported as mean (± standard deviation). Other reported values were collected from a single block of the experiment weekly and are reported as measured values for each treatment.

**Ambient Control Warmed Control Ambient Atrazine Warmed Atrazine**

**Dissolved Oxygen (%)**

May 16^a^  87.5 (13.2) 81.8 (12.8) 93.6 (13.2) 84.6 (8.7)

May 19^b^  102.4 106.0 104.2 89.2

May 26^b^  101.1 94.4 102.3 86.4

May 30^bc^  84.2 89.9 87.0 72.9

June 2^b^ 76.6 81.3 83.0 68.8

June 9^b^ 83.1 91.3 89.2 67.2

June 16^b^ 90.7 85.0 86.1 81.5

June 23^b^ 83.0 91.1 80.1 75.0

June 30^b^ 88.5 96.1 87.5 76.8

July 7^b^ 70.0^de^ 79.8^de^ 56.0^def^ 62.0^de^

July 14^b^ 90.1 91.5 87.8 90.3

**Specific Conductance (µS/cm)**

May 16^a^  274.5 (7.6) 357.3 (8.6) 274.0 (3.6) 349.4 (9.3)

May 19^b^  286.0 366.0 281.7 365.4

May 26^b^  294.1 370.7 876.0 370.5

Table S11: Continued.

**Ambient Control Warmed Control Ambient Atrazine Warmed Atrazine**

May 30^bc^  292.1 373.3 288.5 370.2

June 2^b^ 314.4 391.3 312.1 397.7

June 9^b^ 343.1 417.7 341.3 399.6

June 16^b^ 397.0 463.3 390.1 466.8

June 23^b^ 460.7 532.0 453.3 536.0

June 30^b^ 489.2 568.0 476.5 564.0

July 7^b^ 537.0 622.0 540.0 622.0

July 14^b^ 607.0 680.0 583.0 664.0

**Conductivity (µS/cm)**

May 16^a^  220.5 (9.2) 306.5 (7.2) 219.7 (3.0) 300.3 (6.9)

May 19^b^  221.0 303.4 216.9 302.4

May 26^b^  241.3 326.3 234.0 327.5

May 30^bc^  244.2 327.0 239.4 322.0

June 2^b^ 297.0 376.0 294.0 384.1

June 9^b^ 287.3 365.3 284.7 358.8

June 16^b^ 368.3 451.2 361.3 452.1

June 23^b^ 452.0 546.0 444.2 548.0

June 30^b^ 450.0 539.0 437.2 534.0

July 7^b^ 547.0 633.0 531.0 626.0

Table S11: Continued.

**Ambient Control Warmed Control Ambient Atrazine Warmed Atrazine**

July 14^b^ 546.0 625.0 524.0 605.0

**pH**

May 16^a^  7.38 (0.07) 7.44 (0.06) 7.42 (0.17) 7.35 (0.24)

May 19^b^  7.10 7.24 7.00 6.96

May 26^b^  7.15 7.26 6.81 6.83

May 30^bc^  6.94 7.19 6.88 6.79

June 2^b^ 7.22 7.43 7.06 6.90

June 9^b^ 6.89 7.30 6.76 6.72

June 16^b^ 7.22 7.14 7.04 6.86

June 23^b^ 7.23 7.31 6.98 7.19

June 30^b^ 7.04 7.12 6.92 6.91

July 7^b^ 7.17 7.46 6.88 7.01

July 14^b^ 7.17 7.38 7.03 7.15

**Unionized Ammonia (mg/L)**^g^

May 16^a^  0 (0) 0 (0) 0 (0) 0 (0)

May 19^b^  0 0 0 0

May 26^b^  0 0 0 0

May 30^bc^  0 0 0 0

June 2^b^ 0 0 0 0

Table S11: Continued.

**Ambient Control Warmed Control Ambient Atrazine Warmed Atrazine**

June 9^b^ 0 0 0 0

June 16^b^ 0 0 0 0

June 23^b^ 0.02 0.01 0.01 0.01

June 30^b^ 0.02 0.01 0.01 0.02

July 7^b^ 0.06 0.10 0.02 0.02

July 14^b^ 0.03 0.06 0.02 0.02

**Nitrate (ppm)**

May 16^a^  0 (0) 0 (0) 0 (0) 0 (0)

May 19^b^  0 0 0 0

May 26^b^  0 0 0 0

May 30^bc^  0 0 0 0

June 2^b^ 0 0 0 0

June 9^b^ 0 0 0 0

June 16^b^ 0 0 0 0

June 23^b^ 0 0 0 0

June 30^b^ 5.0 0 2.5 2.5

July 7^b^ 2.5 0 2.5 0

July 14^b^ 0 0 0 0

**Nitrite (ppm)**

Table S11: Continued.

**Ambient Control Warmed Control Ambient Atrazine Warmed Atrazine**

May 16^a^  0 (0) 0 (0) 0 (0) 0 (0)

May 19^b^  0 0 0 0

May 26^b^  0 0 0 0

May 30^bc^  0 0 0 0

June 2^b^ 0 0 0 0

June 9^b^ 0 0 0 0

June 16^b^ 0 0 0 0

June 23^b^ 0 0.25 0 0.25

June 30^b^ 0.25 0.25 0.25 0.25

July 7^b^ 0.25 0 0 0

July 14^b^ 0.50 0 0 0

**Hardness (ppm)**

May 16^a^  46.5 (9.8) 68.0 (8.0) 53.7 (0.0) 68.0 (8.0)

May 19^b^  53.7 71.6 53.7 71.6

May 26^b^  53.7 71.6 53.7 71.6

May 30^bc^  53.7 71.6 53.7 71.6

June 2^b^ 53.7 71.6 53.7 71.6

June 9^b^ 71.6 71.6 53.7 71.6

June 16^b^ 71.6 89.5 71.6 89.5

Table S11: Continued.

**Ambient Control Warmed Control Ambient Atrazine Warmed Atrazine**

June 23^b^ 89.5 107.4 71.6 89.5

June 30^b^ 71.6 107.4 89.5 107.4

July 7^b^ 89.5 107.4 89.5 107.4

July 14^b^ 107.4 107.4 107.4 107.4

**Temperature (°C)**^h^

May 16^a^  14.7 (0.8) 17.6 (0.9) 14.6 (0.6) 17.4 (0.6)

May 19^b^  13.2 16.1 13.0 16.0

May 26^b^  15.7 18.7 15.4 19.0

May 30^bc^  16.4 18.5 16.1 18.1

June 2^b^ 22.1 22.9 22.0 23.2

June 9^b^ 16.5 18.5 16.3 18.3

June 16^b^ 21.3 23.6 21.2 23.3

June 23^b^ 24.0 26.4 24.0 26.2

June 30^b^ 20.8 22.3 20.7 22.2

July 7^b^ 24.5 25.7 24.2 25.4

July 14^b^ 19.7 20.8 19.6 20.3

^a^ Sampled from all treatments across all blocks (N = 20 total).

^b^ Sampled from treatments in replicate block 3 (N = 4 total).

^c^ Date week 2 endpoint collection began.

^d^ Values low as bubblers were removed to look for stage 46 frogs.

^e^ Values low due to faulty bubbler. Bubbler was replaced after measurement.

^f^ Value low because air stone was not in mesocosm properly. Air stone adjusted immediately after measurement.

^g^ Value calculated according to (Francis-Floyd et al., 1990)

^h^ Temperature readings were measured using YSI probe.

Table S12: Table of measured atrazine in rainwater throughout experimental period.

**Date Atrazine Concentration (μg/L)**

June 16^th^ 0.128

June 30^th^ 0.054

July 7^th^ 0.051

July 17^th^ 0.010


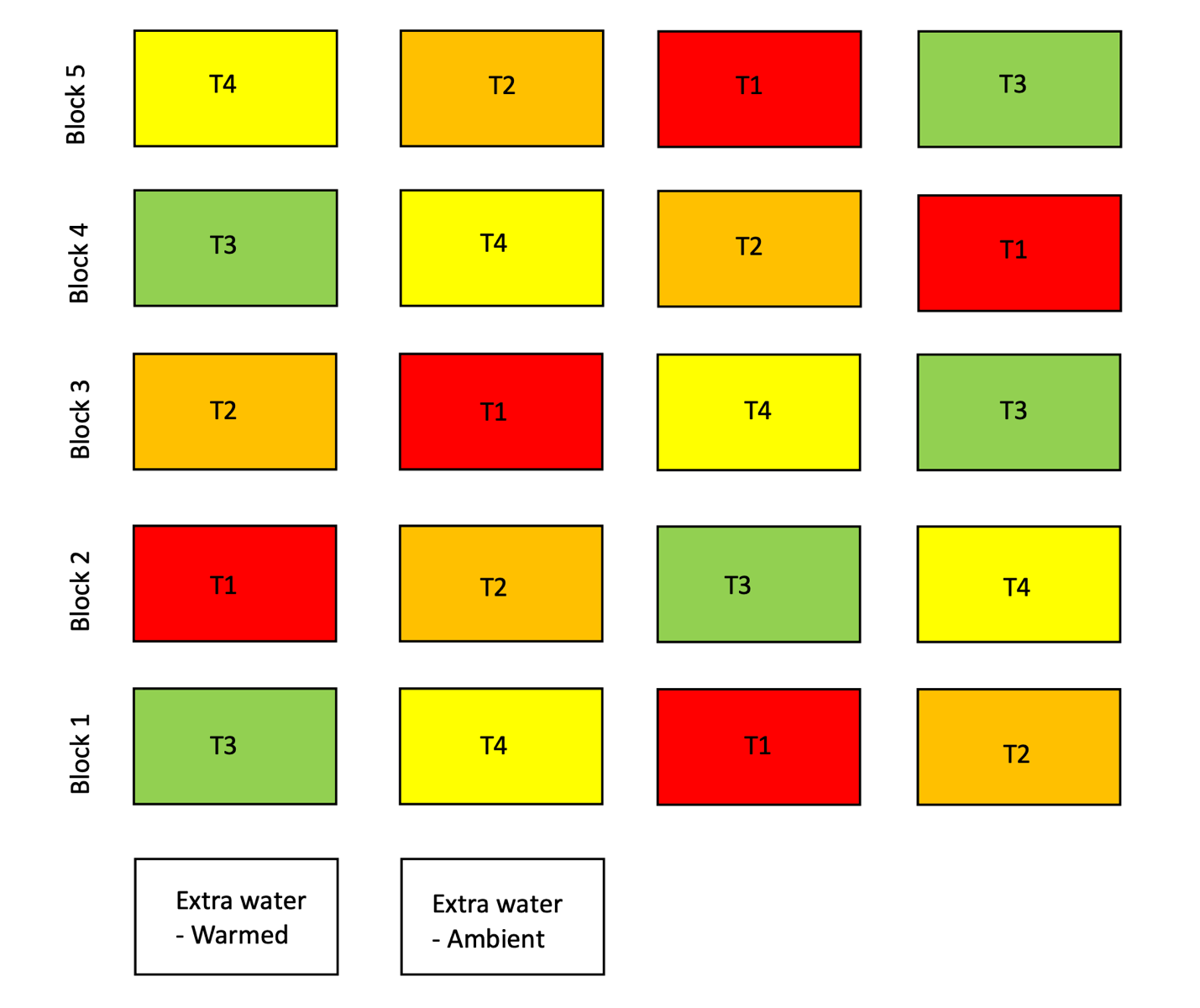


Figure S1: Experimental layout of mesocosms. T1 corresponds to ambient controls, T2 corresponds to warmed controls, T3 corresponds to ambient atrazine, and T4 corresponds to warmed atrazine.


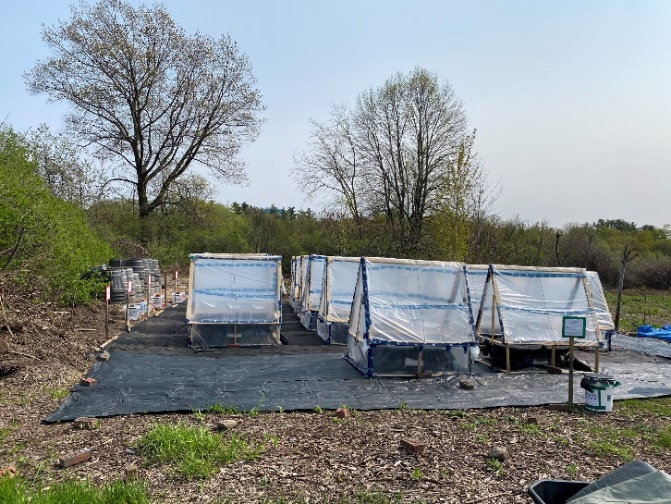

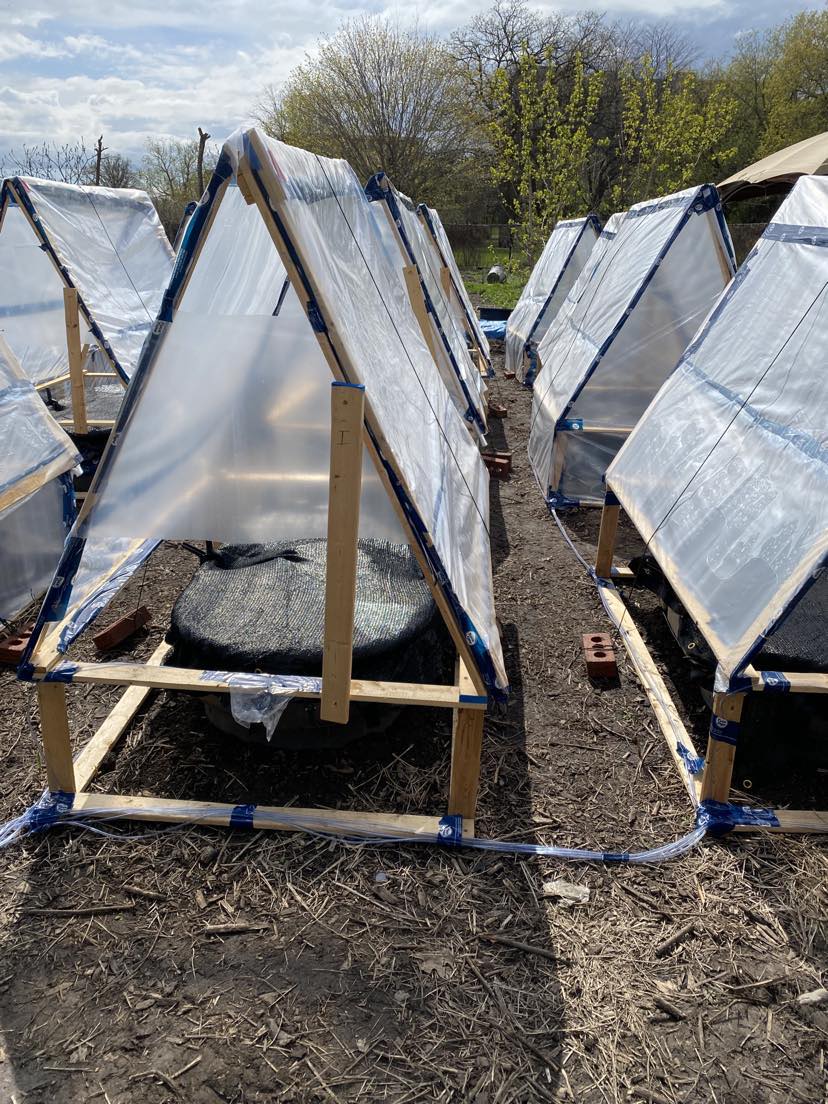


A

B

Figure S2: (A) Greenhouse design used in study. (B) View of vented greenhouses.

B


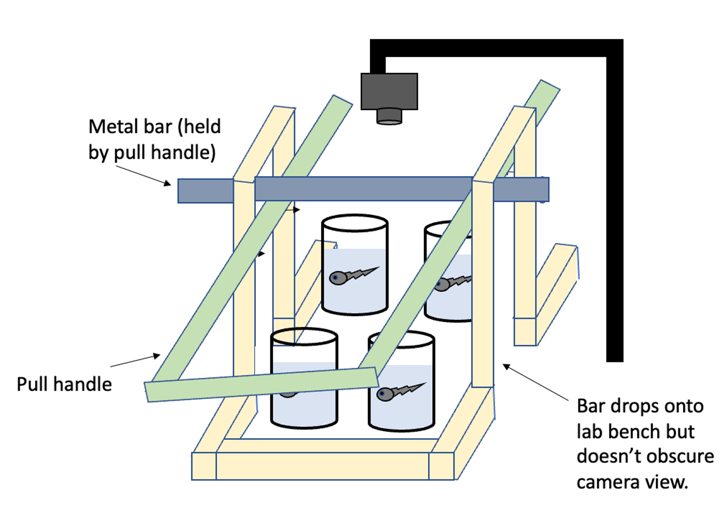

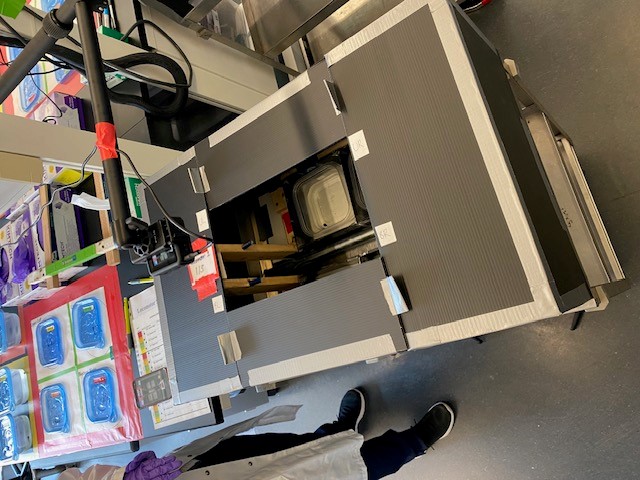


A

Figure S3: (A) Schematic for tadpole standardized startle assay arena. (B) Photo of tadpole standardized startle assay arena.


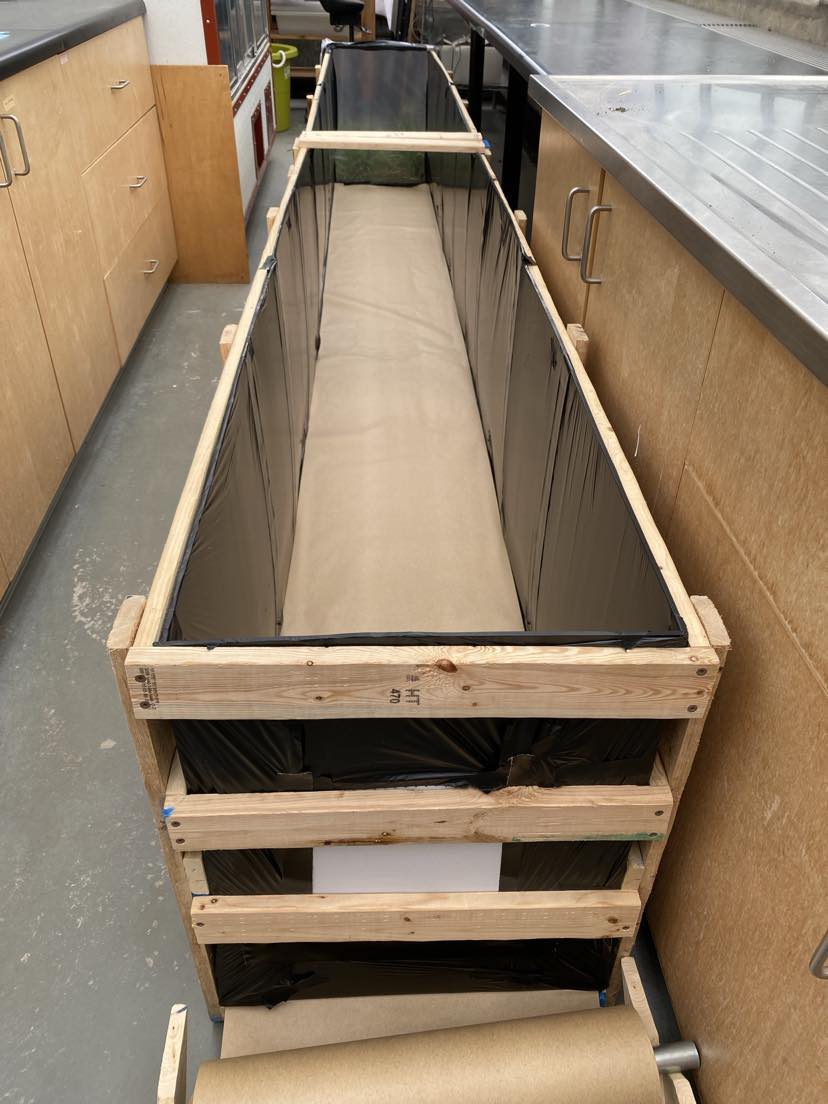


Figure S4: Photo of metamorph locomotor assay arena.


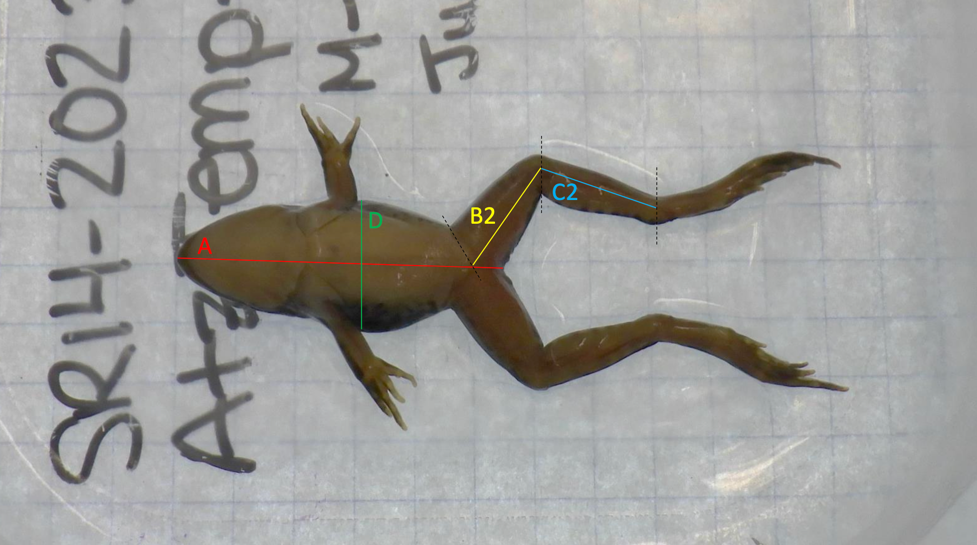


Figure S5: Measurements for (A) Snout to vent length, (B2) Femur length, (C2) Tibiofibular Length, and (D) body width for Gosner stage 46 metamorph measures.


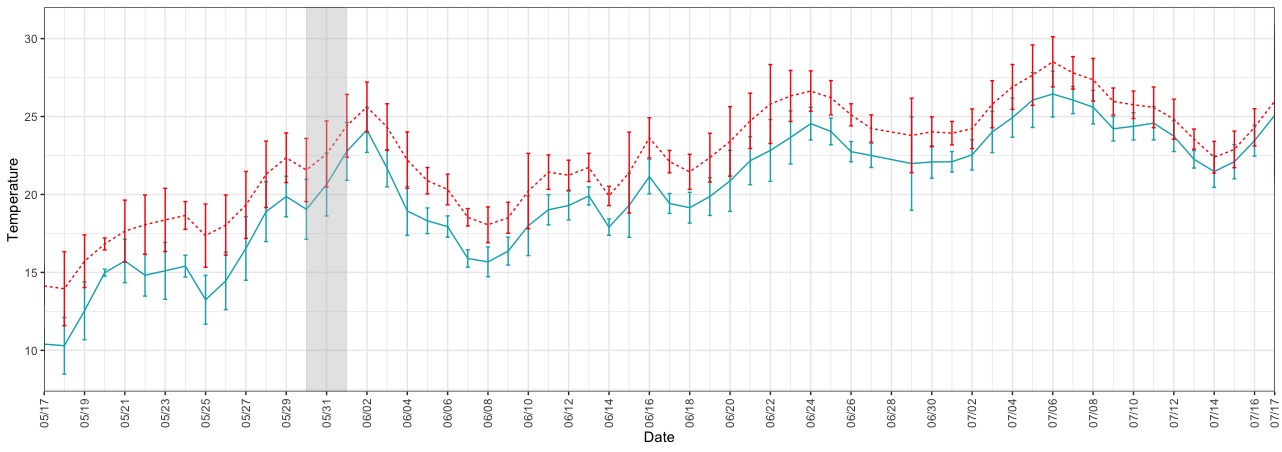


Figure S6: Time series of mean mesocosm temperatures (°C) and standard deviation. The warmed tanks are depicted by the dashed red line, whereas the ambient tanks are depicted by the blue line. The grey bar indicates the dates tadpoles were collected for week two endpoint collection. Mean temperatures were based on hourly temperature readings from HOBO dataloggers.

**References:**

Francis-Floyd, R. Watson, C., Petty, D., & Pouder, D. B. (1990). Ammonia in aquatic systems. *Department of Fisheries and Aquatic Sciences, Florida Cooparerative Extension Service, Institute of Food and Agricultural Sciences, University of Florida, 1 FA.16 University of Florida*.

Heinen, J. T., & Hammond, G. (1997). Antipredator behaviors of newly metamorphosed green frogs (*Rana clamitans*) and leopard frogs (*R. pipiens*) in encounters with eastern garter snakes (*Thamnophis S. Sirtalis*). *American Midland Naturalist, 137*(1), 136. http://doi.org/10.2307/2426762

Zug, G. K. (1975). Anuran locomotion – Structure and function, 2: Jumping performance of semiaquatic, terrestrial and arboreal frogs. In *Smithsonian Contributions to Zoology.* Smithsonian Institution Press.
